# Transcriptomic Insights into the Atrial Fibrillation Susceptibility Locus near the *MYOZ1* and *SYNPO2L* Genes

**DOI:** 10.3390/ijms251910309

**Published:** 2024-09-25

**Authors:** Sojin Y. Wass, Han Sun, Gregory Tchou, Nana Liu, David R. Van Wagoner, Mina K. Chung, John Barnard, Jonathan D. Smith

**Affiliations:** 1 Departments of Cardiovascular & Metabolic Sciences, Cleveland Clinic, Cleveland, OH 44195, USA; 2 Department of Cardiovascular Medicine, Cleveland Clinic, Cleveland, OH 44195, USA; 3 Department of Quantitative Health Sciences, Cleveland Clinic, Cleveland, OH 44195, USA; 4 Department of Molecular Medicine, Cleveland Clinic Lerner College of Medicine, Case Western Reserve University, Cleveland, OH 44195, USA

**Keywords:** *MYOZ1*, *SNYPO2L*, atrial fibrillation, GWAS, eQTL

## Abstract

Genome-wide association studies have identified a locus on chromosome 10q22, where many co-inherited single nucleotide polymorphisms (SNPs) are associated with atrial fibrillation (AF). This study seeks to identify the impact of this locus on gene expression at the transcript isoform level in human left atria and to gain insight into potential causal variants. Bulk RNA sequencing was analyzed to identify myozenin 1 (*MYOZ1*) and synaptopodin 2-like (*SYNPO2L*) transcript isoforms and the association of common SNPs in this region with transcript isoform expression levels. Chromatin marks were used to suggest candidate regulatory SNPs in this region. Protein amino acid changes were examined for predicted functional consequences. Transfection of *MYOZ1* and two *SYNPO2L* isoforms were performed to localize their encoded proteins in cardiomyocytes derived from stem cells. We identified one *MYOZ1* transcript isoform and four *SYNPO2L* transcript isoforms, two of which encode proteins, while the other two encode long noncoding RNAs (lncRNAs). The risk allele of the strongest AF susceptibility SNP on chromosome 10q22 is associated with decreased *MYOZ1* expression and increased expression of the two *SNYPO2L* lncRNA isoforms. There are many SNPs co-inherited with the top AF-associated SNP due to linkage disequilibrium (LD), including rs11000728, which we propose as the *MYOZ1* regulatory SNP, confirmed by reporter gene transfection. In addition, this LD block includes three missense SNPs in the *SYNPO2L* gene, with the minor protective haplotype predicted to be detrimental to protein function. MYOZ1 and both protein isoforms of SYNPO2L were localized to the sarcomere. This is a complex locus with the potential for several SNPs in a haplotype to alter AF susceptibility by opposing effects on *MYOZ1* and *SYNPO2L* lncRNA expression, along with effects on SYNPO2L protein function.

## 1. Introduction

Atrial fibrillation (AF) is the most common sustained arrhythmia, with serious morbidities and associated costs [1]. The projected prevalence of AF is expected to grow with population aging and the global rise in obesity [2,3]. However, our understanding of AF pathogenesis remains elusive, and treatment of AF is limited by unfavorable side effects and widely varying efficacy rates [4]. Understanding genetic mechanisms that underlie AF may help identify novel targets for therapy and disease modification.

Genome-wide association studies (GWAS) have now identified > 100 AF risk loci [5,6,7]. At many loci, the top GWAS single nucleotide polymorphism (SNP), defined as the common genetic variant with the most significant association with the trait, is often intergenic or intronic and does not necessarily reside closest to the causative gene. Also, due to linkage disequilibrium (LD), the top GWAS SNP may not be the causal SNP but may be co-inherited with the causal SNP. Thus, identification of the causal gene and causal SNPs is the first step in understanding how these genetic variants alter AF susceptibility [8]. In many cases, the causal GWAS SNP is regulatory and alters the expression of the causal gene or transcript isoform without altering a codon within the protein coding region of the gene. *MYOZ1* and *SYNPO2L* have been identified as potential AF causal genes because they are close to the top GWAS SNP on chromosome 10q22, and both genes encode proteins that are found on the Z-disc or sarcomeres in cardiomyocytes.

*MYOZ1* encodes myozenin 1, also known as FATZ-1 or calsarcin-2. It is a key Z-disc protein thought to function as a scaffold with multiple binding sites for various sarcomeric partners such as α-actinin, thereby regulating skeletal muscle structure and function. MYOZ1 is implicated in the assembly and stabilization of the Z-discs in striated muscle [9].

*SYNPO2L* encodes synaptopodin 2-like protein, also known as CHAP (cytoskeletal heart-enriched actin-associated protein) that is expressed in striated heart and skeletal muscle cells; it is essential for maintaining the structural integrity and function of muscle fibers [10]. CHAP localizes to the sarcomere and it can also translocate to the nucleus. It is associated with filamentous actin in both locations. Ectopic expression of CHAP in vitro disrupts the subcellular localization of alpha-actinin in rat neonatal cardiomyocytes. Knockdown of CHAP in zebrafish results in abnormal cardiac and skeletal muscle development and function [11]. Misregulated expression of the fetal isoform CHAPb in a mouse model results in cardiomyopathy [12].

In the current study, we aimed to gain more insight into the mechanism for the AF GWAS locus located near the *MYOZ1* and *SYNPO2L* genes. We examined our left atrial appendage RNAseq data in more detail to determine the transcript isoforms of *SYNPO2L*, their protein coding potential, and if their expression levels were associated with the AF risk variant at this locus. We also used bioinformatics and reporter gene transfection to identify the top candidate regulatory variant at this locus.

## 2. Results

### 2.1. RNAseq Analysis and Identification of Transcript Isoform-Specific eQTLs

**Figure 1:**
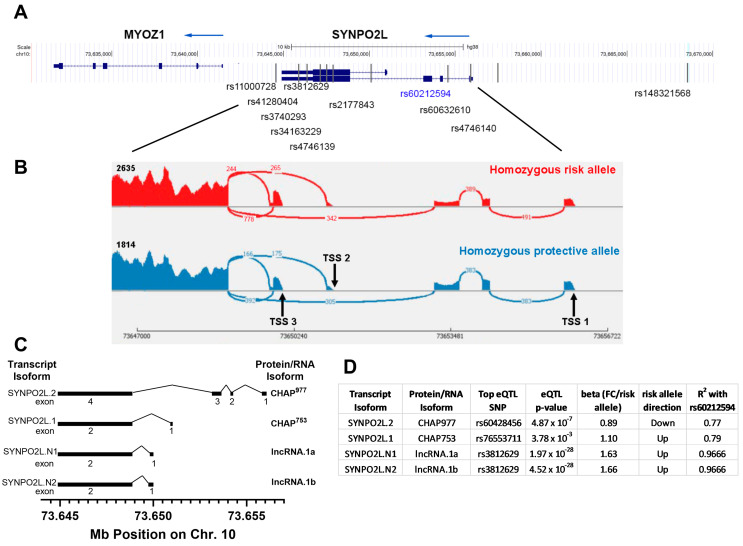
*SYNPO2L MYOZ1* gene locus and transcript isoforms. (**A**) Gene cluster and positions of some relevant SNPs. (**B**) IGV browser Sashimi plot showing LAA RNAseq exon and splice junction reads for one representative subject each homozygous for the risk or protective allele of rs60212594. (**C**) Map of four *SYNPO2L* transcript isoforms, showing three transcription start sites (TSS) and one alternative splice donor yielding two protein coding and two long noncoding RNAs (lncRNA) transcript isoforms. (**D**) Top eQTLs for the four *SYNPO2L* transcript isoforms along with their *p*-values and beta coefficients.

The top AF GWAS SNP at chr 10q22 is rs60212594, where the major allele is the AF risk allele (*p*-value = 6.482 × 10^−27^) [7]. This SNP is the strongest eQTL associated with *MYOZ1* and *SYNPO2L* expression in our left atrial appendage (LAA) RNAseq analysis of 230 European descent subjects. The risk allele is associated with decreased *MYOZ1* expression (*p*-value = 5.62 × 10^−45^) and increased expression of *SYNPO2L* (*p*-value = 3.72 × 10^−14^) [8]. The position of this SNP and its position relative to these genes are shown in Figure 1A. Examples of the IGV browser [13] view of LAA RNAseq from subjects homozygous for the risk and protective alleles are shown in Figure 1B, where the Sashimi plot indicates the number of splice junction reads. There are three transcription start sites (TSS), numbered 1–3, and one alternative splice donor site (from the exon starting at TSS 3).

The resulting four transcript isoforms are shown in Figure 1C. Only two of these isoforms were previously annotated in Ensembl, SYNPO2L-202 (called here SYNPO2L.2) and SYNPO2L-201 (SYNPOL.1). SYNPO2L.2 encodes the full-length isoform (four exons, 977 amino acids (aa), CHAP^977^). SYNPO2L2.1 encodes an N-terminal truncated isoform (two exons with unique first exon, 753 aa, CHAP^753^). We named the other two novel isoforms SYNPO2L.N1 and SYNPO2L.N2; both contain two exons and use TSS 3. Whereas SYNPO2L.N1 exon 1 extends for 200 nucleotides (nt) before splicing to the common last exon, SYNPO2L.N2 exon 1 extends for 280 nt. Both of these novel isoforms appear to be long noncoding RNAs (lncRNAs 1a and 1b, Figure 1C) as their first ATG start codons are followed shortly thereafter by stop codons. The cDNA sequences of SYNPO2L.N1 and SYNPO2L.N2, the positions of their first ATG start codons, and the short amino acid sequence they could encode are shown in Supplemental File S1. We searched for conservation of the novel exon used in SYNPO2L.N1 and only found high similarity (94.5% identity) in two primate species: rhesus and black-and-white snub-nosed monkeys.

**Figure 2:**
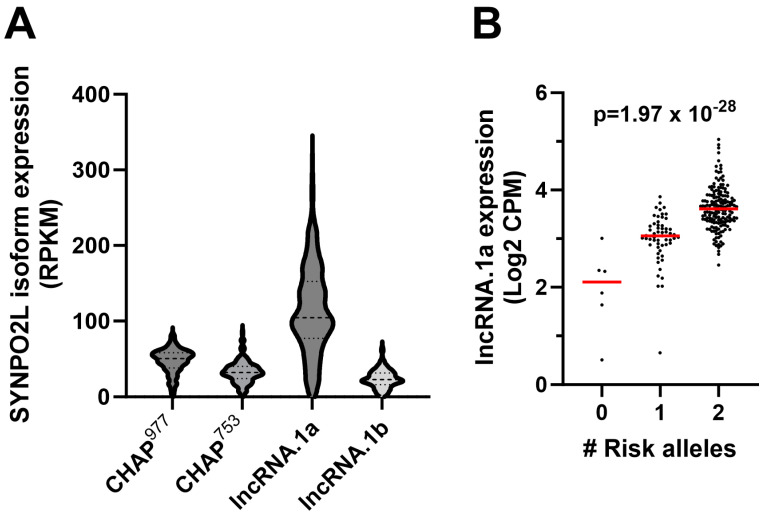
LAA expression of the four *SYNPO2L* isoforms. (**A**) Relative expression levels of the four transcript isoforms in human LAA in RPKM (reads per kilobase per million mapped reads). (**B**) The effect of the AF GWAS risk allele of rs60212594 on the expression of the SYNPO2L.N1 isoform encoding lncRNA.1a (log2 counts per million, *p*-value = 1.97 × 10^−28^ by linear regression adjusted for surrogate variables, age, and sex, red line denotes median value).

The top eQTL SNP for the SYNPO2L.2 transcript encoding the canonical CHAP^977^ protein was rs60428456 (Figure 1D). The top eQTL SNP for SYNPO2L.1 encoding CHAP^753^ was rs76553711. The top eQTL for both of the novel lncRNA transcript isoforms was rs3812629, which is in very high LD with the top GWAS SNP (Figure 1D). However, the direction and strength of these top eQTLs differ among transcript isoforms. The risk allele of the top eQTL SNP is associated with lower expression of the full-length SYNPO2L.2 isoform (*p* = 4.87 × 10^−7^), and higher expression levels of the SYNPO2L.1 isoform (*p* = 3.87 × 10^−3^). The strength of these eQTLs is dwarfed by the strength of the eQTLs for the two novel lncRNA isoforms, where the risk allele is strongly associated with higher expression of SYNPO2L.N1 (*p* = 1.97 × 10^−28^) and SYNPO2L.N2 (*p* = 4.52 × 10^−28^). Thus, the gene-level SYNPO2L eQTL for rs60212594 is largely driven by expression of the two novel lncRNA isoforms. The relative expression levels of the four major transcript isoforms in human LAA are shown in Figure 2A, where all isoforms are expressed with median expression levels between 23 and 104 RPKM (reads per kilobase per million mapped reads). The SYNPO2L.N1 isoform encoding lncRNA.1a is expressed most robustly, and the effect of the AF risk allele of rs60212594 on its expression level in human LAA is shown in Figure 2B.

### 2.2. Identification of Putative Regulatory SNPs Controlling MYOZ1 and SYNPO2L Expression

**Figure 3:**
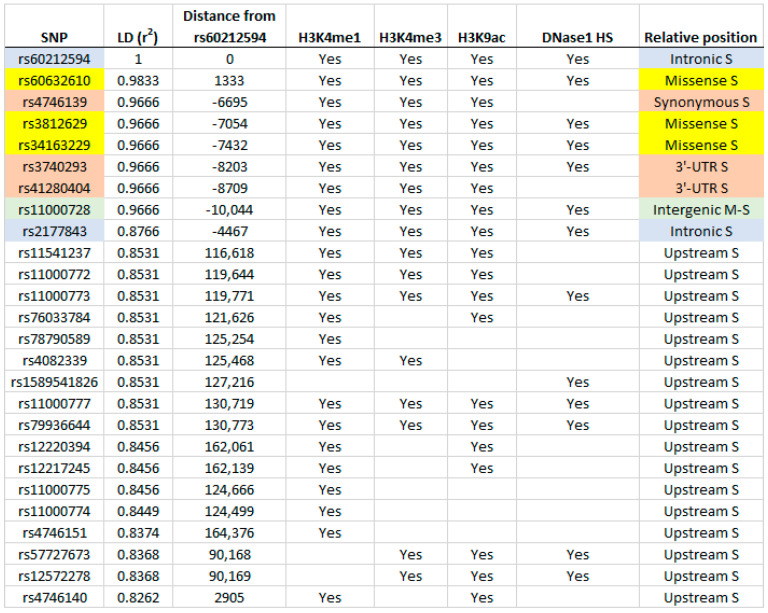
Candidate regulatory and missense SNPs in linkage disequilibrium with rs60212594. Missense SNPs (highlighted in yellow); *SYNPO2L* intronic SNPs (highlighted in blue); intergenic SNP between the *MYOZ1* and *SYNPO2L* genes (highlighted in green); synonymous SNPs in the coding region or in the 3′ UTR (highlighted in pink). Regulatory marks include histone 3 lysine 4 mono methylation and trimethylation (H3K4me1 and H3K4Me3), histone 3 lysine 9 acetylation (H3K9ac), and DNase1 hypersensitivity (DNase1 HS). Relative positions to SYNPO2L gene (S) and MYOz2 gene (M).

We assembled data for the 68 SNPs in high LD with rs60212594 (r^2^ ≥ 0.80) with their eQTL −log10 *p*-values and beta coefficients for the gene-level expression of MYOZ1 and SYNPO2L (Supplemental File S2). For each SNP, chromatin conformation was evaluated in human fetal heart tissue using HaploReg V4.2 [14] supplemented with data from the Epigenome roadmap [15]. Of the 68 SNPs, 13 SNPs are in regions of DNase1 hypersensitivity (Figure 3) and 26 are within peaks of enhancer and/or promoter marks, using H3K4me1, H3K4me3, or H3K9ac (Figure 3). Among these putative regulatory SNPs are three *SYNPO2L* missense SNPs (highlighted in yellow), which are inherited as a haplotype block along with the GWAS SNP. rs60632610 alters the second amino acid residue from glycine (major risk allele) to serine (G2S). The glycine residue is highly conserved and PolyPhen2 predicts this as probably damaging to protein function (score 0.997 on a scale from 0, not damaging, to 1, likely damaging). rs3812629 alters residue 707 going from proline to leucine (P707L) within a proline-rich repeated sequence (KTPPP), which also disrupts a highly conserved residue, leading to a probably damaging score of 1.000. rs34163229 alters residue 833 going from serine to tyrosine (S833Y), again probably damaging with a score of 0.956. CHAP^977^ contains all three of these missense SNPs, while CHAP^753^ contains only the latter two missense SNPs. Two putative regulatory SNPs are intronic to *SYNPO2L* (Figure 3, highlighted in blue), while rs11000728 is the only high LD SNP between the *MYOZ1* and *SYNPO2L* genes (highlighted in green). Highlighted in pink are the three SNPs within the *SYNPO2L* gene synonymous either in the coding region or in the 3′ UTR. The positions of these highlighted SNPs are shown in the genome map in Figure 1A.

**Figure 4:**
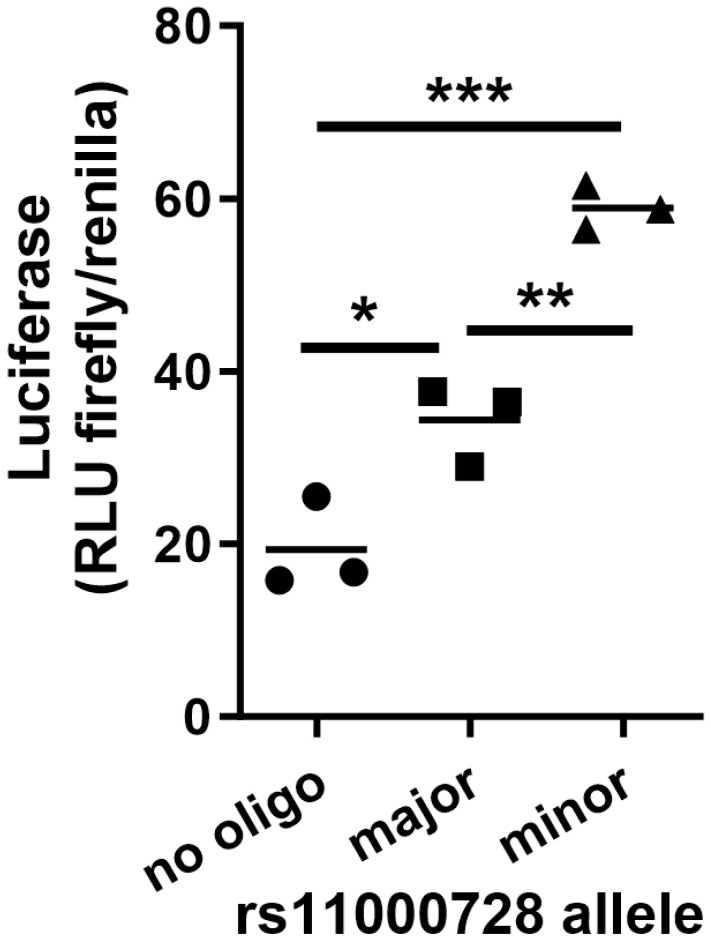
Dual luciferase reporter gene transfection in iCMs with plasmids containing 30-mer oligos surrounding rs110000728. Enhancer activity was observed for both alleles but was ~2-fold weaker for the major/risk allele concordant with its effect on the LAA expression of MYOZ1. * *p* < 0.05; ** *p* < 0.01; *** *p* < 0.001.

We hypothesized that rs110000728 regulates expression of *MYOZ1* since it is the only intergenic SNP between *MYOZ1* and *SYNPO2L* in this LD block, and it is located in a region with DNase1 hypersensitivity and enhancer/promoter marks. Dual luciferase reporter gene transfection in induced pluripotent stem cell-derived cardiomyocytes (iCMs) with plasmids containing 30-mer oligos surrounding rs110000728 revealed enhancer activity for both alleles, but the enhancer activity was ~twofold weaker for the major/risk allele (Figure 4), concordant with the major/risk allele associated with lower *MYOZ1* mRNA expression in human LAA. The SNPs regulating the expression levels of the four *SYNPO2L* transcript isoforms have not been identified, but they are likely to be contained in Figure 3.

### 2.3. Transient Expression of Fluorescently Tagged MYOZ1 and SYNPO2L Isoforms in iCMs

**Figure 5:**
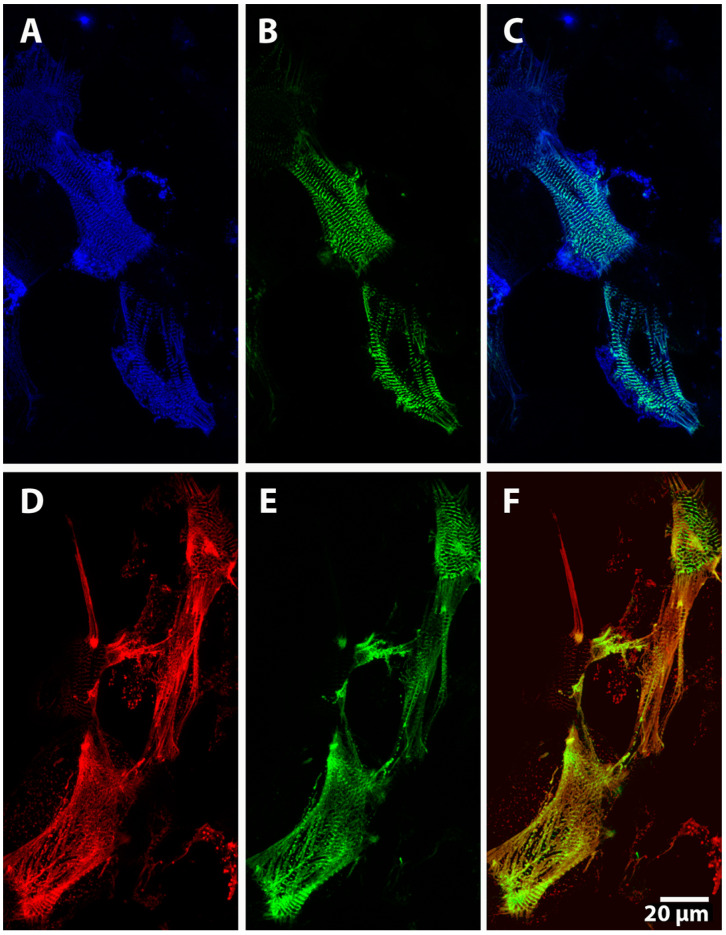
Localization of CHAP isoforms and MYOZ1 to the sarcomere. Co-transfection of SYNPO2L.2/CHAP^977^-BFP fusion protein (**A**) and MYOZ1-EGFP fusion protein (**B**) into iCell cardiomyocytes, along with merged image (**C**). Co-transfection of SYPO2L.1/CHAP^753^-mApple fusion protein (**D**) and MYOZ1-EGFP (**E**) into iCell cardiomyocytes, along with merged image (**F**). 63× objective lens; scale bar, 20 µm.

We designed expression vectors driven by the ubiquitously expressed EF1ꭤ promoter to transiently transfect human MYOZ1 and both SYPO2L/CHAP isoforms with C-terminal fusions to various fluorescent proteins. MYOZ1-EGFP, CHAP^977^-mBFP2 (blue fluorescent protein (FP)), and CHAP^753^-mApple were transiently transfected into iCell cardiomyocytes. MYOZ1-GFP colocalized with both CHAP isoforms in the sarcomeres (Figure 5).

### 2.4. Left Atrial Coexpression with SYNPO2L or MYOZ1

Coexpression analysis from LA RNAseq demonstrated that the top gene correlated with *MYOZ1* expression was an inverse correlation with *SYNPO2L* (R = −0.52; adj *p*-value = 7.71 × 10^−11^). Conversely, the top gene correlated with *SYNPO2L* expression was an inverse correlation with *MYOZ1* (R = −0.50; adj *p*-value = 1.90 × 10^−9^). The lists of *MYOZ1* and *SYNPO2L* coexpressed genes at an unadjusted *p*-value < 0.001 are shown in Supplemental Files S3 and S4. Table 1 shows the gene set enrichment analysis results identifying the top hallmark pathways (*p* < 0.05) from the *MYOZ1* and *SYNPO2L* coexpression data. *MYOZ1* coexpression included positive associations with cholesterol homeostasis, uv response up, MTORC1 signaling, and DNA repair pathways. *MYOZ1* coexpression included inverse associations with oxidative phosphorylation, epithelial-to-mesenchymal transition, and uv response down pathways. *SYNPO2L* coexpression included positive associations with oxidative phosphorylation, myogenesis, adipogenesis, fatty acid metabolism, apical junction, uv response down, and epithelial-to-mesenchymal transition pathways. *SYNPO2L* coexpression included inverse associations with cholesterol homeostasis, interferon alpha response, MTORC1 signaling, interferon gamma response, spermatogenesis, and DNA repair pathways. Thus, many of these top pathways were reciprocally associated with *MYOZ1* and *SYNPO2L* coexpression.

## 3. Discussion

An AF GWAS from 2018 identified rs60212594 as the top AF-associated SNP at chr 10q22 [6]. This SNP is located in the first intron of the canonical four-exon SYNPO2L mRNA. Our LAA RNAseq study identified rs60212594 as the top eQTL SNP associated with the gene-level expression of both the *MYOZ1* and *SYNPO2L* genes, with the risk variant associated with increased *SYPO2L* and lower *MYOZ1* expression [8]. However, here, we report that there are four distinct SYNPO2L mRNA isoforms, originating from three transcription start sites. Two of these isoforms are novel, encoding lncRNAs that were not identified in Ensembl. We found that the gene-level *SYNPO2L* eQTL is driven largely by the expression of these two novel noncoding isoforms. LncRNAs can act at different levels via epigenetics or other mechanisms to regulate gene expression in cis or in trans [16]. We do not know the function of the two SYNPO2L lncRNAs, but it is tempting to speculate that they might decrease expression of the adjacent *MYOZ1* gene and be partially responsible for their inverse coexpression. Similarly, we previously characterized the PANCR lncRNA in human LAA, which regulated the expression of the adjacent *PITX2* gene, although in this case, it increased the expression of the adjacent gene [17]. Since the expression of the SYNPO2L lncRNAs are most robustly associated with the AF risk haplotype, more research is required to determine if there is a mechanistic link between these lncRNAs and AF pathogenesis in humans.

It is intriguing that the sequence of the exons unique to the SYNPO2L lncRNAs is conserved only in two primate species, rhesus and snub-nosed monkeys, but not in other primates like chimpanzees that are more closely related to humans. It seems unlikely that this sequence was inserted two or more distinct times into primates during evolution. There are two potential explanations for this observation. Either the lncRNA sequence evolved before the branching of humans from rhesus and snub-nosed monkeys, and this sequence was lost during evolution of most primate species aside from those three species, or there was horizontal gene transfer between primate species into the human lineage and not into more closely related primates.

In mice, two CHAP protein isoforms (CHAPa, 978 aa residues, and CHAPb, 749 aa) have been characterized, similar to the human CHAP^977^ and CHAP^753^ isoforms. Van Eldik et al. showed that the shorter isoform is predominant in the fetal mouse heart, while the longer isoform becomes predominant in the adult mouse heart [12]. Changing the ratio of the isoforms in a transgenic mouse model, in which the shorter fetal isoform became the predominant isoform in the adult mouse heart, resulted in enlarged atria, cardiac hypertrophy, contractile dysfunction, impaired sarcomere function and organization, fibrosis and stress fiber formation [12]. Whether the ratio of the human CHAP^977^ and CHAP^753^ isoforms changes during development is not known. However, the AF risk haplotype is modestly associated with lower expression of CHAP^977^ and higher expression of CHAP^753^, which is in the same direction as the mouse model above. Thus, the expression levels of these two CHAP isoforms may also play a role in AF pathogenesis.

The risk allele rs60212594 is associated with decreased expression of *MYOZ1*. MYOZ1 is crucial for maintaining the structural integrity of the Z-line in muscle cells, including cardiac myocytes [9]. In ducks, MYOZ1 has a positive regulatory effect on muscle growth and development [18]. It binds to α-actinin-2/-3, myotilin, and filamin-C, creating an interaction hub for Z-line proteins and stabilizing the Z-disc [9,19]. Decreased MYOZ1 expression can lead to Z-line disorganization, compromising the structural integrity of cardiac muscle cells. In addition, MYOZ1 interacts with calcineurin. In skeletal muscle, calcineurin activation has been shown to be necessary for hypertrophic growth in response to insulin-like growth factor-1, which can mobilize intracellular calcium [20]. Calcineurin is a key player in the calcineurin–NFAT signaling pathway, which is important for cardiac hypertrophy and muscle differentiation [21]. MYOZ1 inhibits calcineurin/NFAT signaling by sequestering calcineurin to the Z-line, preventing NFAT from translocating to the nucleus and activating target genes [22]. Loss of NFAT activity in the heart results in a deficiency in mitochondrial energy metabolism required for cardiac morphogenesis and function [23].

MYOZ1 is also a target of the circadian E3 ligase FBXL21 [24]. Proper circadian regulation ensures the rhythmic expression and degradation of MYOZ1, maintaining muscle homeostasis [25,26,27]. Deficiency in FBXL21 leads to Z-line disorganization, NFAT inhibition, and impaired muscle differentiation, contributing to atrial structural remodeling [24]. GSK-3β phosphorylates MYOZ1, promoting its degradation [24,28]. GSK-3β coexpression and inhibition were found to accelerate and decelerate FBXL21-mediated MYOZ1 degradation, respectively [24]. Excessive degradation leading to reduced MYOZ1 may disrupt normal cardiac myocyte function.

It is possible that decreased MYOZ1 can also affect the mechanical properties of atrial myocytes, leading to abnormal stretch and strain responses. This may contribute to the electrical remodeling seen in AF, characterized by altered ion channel expression and function [29]. Electrical remodeling further destabilizes atrial myocyte excitability, increasing susceptibility to AF [29]. Inflammation and fibrotic changes alter the extracellular matrix and cellular environment, further disrupting the structural and functional integrity of atrial tissue [30]. Reduced MYOZ1 may contribute to fibrosis and chronic inflammation, common features in AF. We speculate that decreased MYOZ1 expression leads to structural disorganization, impaired calcineurin–NFAT signaling, disrupted circadian regulation, mechanical and electrical remodeling, and fibrosis. These changes may collectively contribute to the pathogenesis of atrial fibrillation by destabilizing the atrial myocytes and increasing susceptibility to arrhythmias.

In addition to examining the role of *MYOZ1* and *SYNPO2L* transcript isoforms on AF susceptibility, we were also interested in identifying the causal variants at this locus. Analysis of the GWAS SNP rs60212594, and those in high LD with it, suggested that the regulatory SNP for *MYOZ1* expression may be rs11000728, based on fetal heart enhancer/promoter and DNaseI hypersensitivity data, and that it is the only high LD SNP located in the intergenic region between *MYOZ1* and *SYNPO2L*. This was further confirmed by the presence of allele specific enhancer activity in reporter gene transfections. The major risk allele of rs11000728 vs. the minor protective allele demonstrated weaker enhancer activity by approximately twofold, consistent with the association of AF risk alleles at this locus with decreased expression of *MYOZ1*. Since the expression of MYOZ1 and the SYNPO2L isoforms is inversely regulated by the AF risk alleles at this locus, we think it is likely that there is a risk haplotype consisting of two or more linked SNPs that decreases *MYOZ1* expression (rs11000728) and regulates the varying expression of the four *SYNPO2L* isoforms. The AF protective haplotype at this locus is not only associated with altered expression of *MYOZ1* and *SYNPO2L* but also leads to a full-length CHAP^977^ protein with three missense variants that are each predicted to damage protein function based upon conservation between species and dissimilarity of the amino acid replacement. Thus, less of a functional SYNPO2L/CHAP protein appears to be protective against AF. However, it is also possible that these three missense variants could lead to a gain of function for CHAP protein with new attributes, which could protect against AF.

### Limitations and Clinical Implications

The RNAseq analysis was performed on European ancestry subjects only. The findings related to eQTLs may not capture the genetic diversity that may be present in other populations or be generalizable across diverse ancestries. In addition, further functional assays are necessary to validate whether these SNPs and different isoforms lead to a meaningful increase in disease risk. Lastly, RNAseq analysis was performed from tissue of the left atrial appendage which may not reflect gene expression changes in the remaining left atrium.

The inverse correlation between *MYOZ1* and *SYNPO2L* expression could indicate a regulatory relationship between these genes in atrial tissue. Therapies that modulate the balance between these two genes could be explored as treatment strategies for AF, such as reducing AF burden. In addition, patients carrying the risk alleles for AF may benefit from early interventions, lifestyle modifications, or tailored therapies aimed at modulating gene expression.

## 4. Materials and Methods

### 4.1. RNAseq and Identification of Transcript Isoform-Specific eQTL Analysis

Cis eQTL SNPs associated with expression of *MYOZ1* and *SYNPO2L* at the gene level were identified in our prior RNAseq study (afeqtls.lerner.ccf.org, accessed on 1 April 2024), from 230 European ancestry LAA human tissues [1]. Additional analyses of our LAA RNAseq study were used to identify the major transcript isoforms of *MYOZ1* and *SYNPO2L,* and the top eQTL SNPs for each transcript isoform.

Identification of each transcript isoform of *SYNPO2L* was performed by examination of the aggregated RNAseq BAM files [31] using the IGV browser [13] combined with transcript annotations from GENCODE and CHESS 3 [32]. A *SYNPO2L* exon that was highly and consistently expressed in our LAA data was not annotated in the GENCODE human transcriptome used for alignment (release 27 or later releases), nor did it match the exon boundaries in CHESS 3. These omissions led us to quantify SYNPO2L transcripts using their most 3′ unique exons as delineated in the aggregated BAM files from our LAA tissues rather than using annotated transcript models.

Specifically, we quantified expression of each SYNPO2L isoform by counting the reads for the most 3′ isoform-unique exonic regions using the summarizeOverlaps function from the R package GenomicAlignments [33] applied to the BAM files produced by the STAR aligner. For any exons that overlapped between isoforms, we calculated reads in the disjoint regions and combined regions, then used these to calculate the deconvolved counts accounting for region lengths. To enable useful visualizations of isoform expression intensities, we then corrected the exon counts for the length of each exon to derive exon intensities in reads per kilobase per million mapped reads (RPKM).

*SYNPO2L* cis SNPs were obtained from Illumina microarrays as previously described [8] combined with imputation using the Haplotype Reference Consortium reference haplotypes [34]. All variants within 500 kilobases of the maximal transcription start and end sites of *SYNPO2L* as defined in GENCODE release 38 with imputation quality greater than 0.8 and minor allele frequency in the LAA set greater than 0.05 were considered in eQTL analyses.

Cis-expression quantitative trait loci (eQTL) analysis for each *SYNPO2L* transcript isoform was performed using the limma-voom regression approach [35] with TMM (trimmed mean of M-values) sample normalization [36] and voom sample weights calculated using all expressed genes. Normalized, weighted, and transformed *SYNPO2L* isoform-specific exon counts were regressed against *SYNPO2L* cis SNPs, where SNPs were encoded as dosages, sex, and 22 surrogate variables (calculated using all genes) [37]. Coefficient estimates from SNP dosages were extracted and tested against the null hypothesis of no association.

### 4.2. Protein Isoform Analysis

To determine whether the SYNPO2L missense variants were likely to have detrimental effects on protein function we used PolyPhen-2, a tool based on species conservation and amino acid similarity, using a scale from 0 (not damaging) to 1 (likely damaging) [38].

### 4.3. Identification of Putative Regulatory Regions

SNPs in high LD (r^2^ > 0.8) with the AF GWAS SNP rs60212594 in all five European descent groups (CEU, TSI, FIN, GBR, IBS, 1000 genomes phase 3, hGR38 high coverage) within a 200 Kb window, were identified using LDlink [39]. We then queried the SNPs using the HaploReg V4.2 website [14] to search human fetal heart tissue for DNAse1 hypersensitivity and CHIPseq evidence of enhancer/promoter histone marks [14].

### 4.4. Reporter Gene Transfection of Human Pluripotent Stem Cell-Derived Cardiomyocytes

Induced human pluripotent stem (iPS) cells were obtained from ATCC (# BXS0116). iPS cells were plated in tissue culture dishes coated with matrigel and differentiated into atrial cardiomyocytes (iCMs) using the method of Burridge et al. [40] with the addition of retinoic acid (1 μM final concentration, added on differentiation days 4, 6, and 8).

To test a putative regulatory SNP, 30-mer double-stranded oligonucleotides of both alleles with HindIII and SalI overhangs centered around SNPs of interest were subcloned into HindIII- and SalI-digested pGL3 (Promega, Madison, WI, USA), a firefly luciferase expression vector driven by a minimal SV40 promoter. The resulting reporter plasmids were co-transfected with Renilla luciferase reporter plasmid (to control for transfection efficiency) into 800,000 iCMs via electroporation (Nucleofector-II program A-23, Lonza, Walkersville, MD, USA, Cat#VPH-5012) and subsequently plated into 24-well plates. Luciferase activity was measured 48 h post transfection with the Dual-Luciferase^®^ ReporterAssay (Promega, Madison, WI, USA) as per the manufacturer’s protocol. Luciferase activity was reported as relative luciferase units (firefly luciferase/Renilla luciferase).

### 4.5. Protein Localization of Tagged MYOZ1 and CHAP Isoforms

EF1α promoter-driven expression vectors for MYOZ1 and the two protein coding transcript isoforms of SYNPO2L, all fused to fluorescent protein tags at the C-terminus, were constructed by VectorBuilder. Constructs were transiently transfected using ViaFect transfection reagent (Promega) into iCell cardiomyocytes (FujiFilm Cellular Dynamics Inc., Madison, WI, USA), on gelatin-coated glass-bottom tissue culture plates. Finally, 72 h after transfection, cells were fixed with 10% phosphate-buffered formalin for 15 min at 37 °C, and cell localization was examined using confocal microscopy.

### 4.6. Statistical Methods

GraphPad Prism was used to assess normality and to perform simple statistical analyses. For the reporter gene transfection study, data were analyzed by two-tailed ANOVA followed by Tukey’s multiple comparison posttest.

## 5. Conclusions

The AF risk locus on chromosome 10q22 is composed of 68 common SNPs that are in high LD (r^2^ > 0.8) with the top GWAS SNP, and thus inherited as a risk haplotype. This is an extremely complex locus, with two genes (MYOZ1 and SYNPO2L) regulated inversely by the AF risk haplotype, four transcript isoforms for one of these genes (SYNPO2L), two of which produce different protein isoforms (CHAP^977^ and CHAP^753^) and two of which produce lncRNAs. In addition, the AF risk haplotype includes three missense variants in SYNPO2L. Although we identified rs11000728 as the likely causal regulatory SNP that alters expression of MYOZ1, it is likely that additional SNPs in the risk haplotype, yet to be verified, regulate the expression of the SYNPO2L transcript isoforms.

## Figures and Tables

**Table 1:** Top hallmark pathways (*p* < 0.05) from gene set enrichment analysis of coexpression data.

**Positive association with MYOZ1 coexpression**		
Rank	Hallmark	*p*-value
1	HALLMARK_CHOLESTEROL_HOMEOSTASIS	2.24 × 10^−2^
2	HALLMARK_UV_RESPONSE_UP	3.09 × 10^−2^
3	HALLMARK_MTORC1_SIGNALING	3.39 × 10^−2^
4	HALLMARK_PI3K_AKT_MTOR_SIGNALING	3.55 × 10^−2^
5	HALLMARK_DNA_REPAIR	4.07 × 10^−2^
**Negative association with MYOZ1 coexpression**		
Rank	Hallmark	*p*-value
1	HALLMARK_OXIDATIVE_PHOSPHORYLATION	8.13 × 10^−3^
2	HALLMARK_APICAL_SURFACE	2.95 × 10^−2^
3	HALLMARK_EPITHELIAL_MESENCHYMAL_TRANSITION	3.39 × 10^−2^
4	HALLMARK_UV_RESPONSE_DN	4.79 × 10^−2^
**Positive association with SYNPO2L coexpression**		
Rank	Hallmark	*p*-value
1	HALLMARK_OXIDATIVE_PHOSPHORYLATION	1.20 × 10^−3^
2	HALLMARK_MYOGENESIS	2.69 × 10^−3^
3	HALLMARK_ADIPOGENESIS	1.55 × 10^−2^
4	HALLMARK_FATTY_ACID_METABOLISM	2.82 × 10^−2^
5	HALLMARK_APICAL_JUNCTION	3.39 × 10^−2^
6	HALLMARK_UV_RESPONSE_DN	3.47 × 10^−2^
7	HALLMARK_EPITHELIAL_MESENCHYMAL_TRANSITION	3.98 × 10^−2^
8	HALLMARK_NOTCH_SIGNALING	4.37 × 10^−2^
9	HALLMARK_COAGULATION	4.47 × 10^−2^
10	HALLMARK_KRAS_SIGNALING_DN	4.79 × 10^−2^
**Negative association with SYNPO2L coexpression**		
Rank	Hallmark	*p*-value
1	HALLMARK_CHOLESTEROL_HOMEOSTASIS	1.20 × 10^−2^
2	HALLMARK_INTERFERON_ALPHA_RESPONSE	1.48 × 10^−2^
3	HALLMARK_MTORC1_SIGNALING	1.66 × 10^−2^
4	HALLMARK_INTERFERON_GAMMA_RESPONSE	2.57 × 10^−2^
5	HALLMARK_SPERMATOGENESIS	3.16 × 10^−2^
7	HALLMARK_P53_PATHWAY	3.55 × 10^−2^
6	HALLMARK_DNA_REPAIR	3.55 × 10^−2^
8	HALLMARK_TNFA_SIGNALING_VIA_NFKB	3.55 × 10^−2^
9	HALLMARK_ALLOGRAFT_REJECTION	3.63 × 10^−2^
10	HALLMARK_INFLAMMATORY_RESPONSE	3.80 × 10^−2^
11	HALLMARK_MYC_TARGETS_V1	3.89 × 10^−2^

## Data Availability

The data presented in this study are available from the corresponding author upon reasonable request.

## Supplementary Materials

The following supporting information can be downloaded at: https://www.mdpi.com/article/10.3390/ijms251910309/s1.
