# Supplementary material for: Transcriptomic Insights into the Atrial Fibrillation Susceptibility Locus near the MYOZ1 and SYNPO2L Genes
Source: Int J Mol Sci. 2024 Sep 25;25(19):10309. doi: 10.3390/ijms251910309 (PMC11477451; doi:10.3390/ijms251910309)
Supplement: Supplementary file 1 [file ijms-25-10309-s001.zip › ijms-3138533-Supplementary Files S1-S4.pdf]

Supplemental File S1.

SYNPO2L.N1 cDNA

First exon highlighted in yellow, second exon highlighted in blue, ATG start codon highlighted in green.

CCTGGCTGGCAGTGGCTGCCTGAGCTCTCTACGTAGAGACAGTTCTGACCCCTGAAGAAGCCCACCCCAC
TACCATCCCCAGCTTGGGGTAAATAATATATTTAGCTGGCGACCTTGAGGTATCCTAAAGATTTGGAGTG
TTGCCTTGGAGAACAACACTCTAACTTTTCCTGCATCTTGGATGGAGACTACCCCTGACTAACTGCAACG
TGCAGAGAGCCTCCAAGAGAAGAGCATAAAAGAGGCCAAGACCAAATGCAGGACAATTGCATCCCTGCTC
ACTGCAGCCCCCAACCCCCACTCCAAAGGGGTACTTATGTTTAAGAAACGGCGGCAGAGAGCCAAGAAGT
ACACCCTGGTGAGCTTCGGGGCTGCTGCTGGGACAGGCGCTGAGGAGGAGGACGGCGTTCCCCCCACGAG
TGAGTCCGAGCTGGACGAAGAAGCCTTCTCTGACGCCCGCAGCCTCACCAATCAATCTGACTGGGACAGT
CCCTATCTGGACATGGAGCTTGCCAGGGCGGGCTCAAGAGCATCAGAGGGCCAGGGCTCTGGGCTGGGAG
GGCAGCTGAGTGAGGTCTCTGGGCGAGGGGTGCAGCTCTTTGAACAGCAGCGCCAGCGCGCAGACTCCAG
CACCCAGGAACTGGCACGGGTCGAACCAGCAGCCATGCTCAACGGGGAAGGCCTGCAGTCACCACCTCGG
GCCCAGAGTGCTCCCCCAGAGGCAGCTGTGCTCCCACCCAGCCCCTTGCCGGCGCCTGTAGCCAGCCCCA
GACCCTTCCAACCAGGTGGTGGAGCCCCGACCCCAGCTCCAAGCATCTTTAACCGGTCAGCCAGGCCCTT
TACCCCGGGCCTACAAGGGCAGCGGCCAACTACCACCTCGGTTATTTTCCGGCCTTTAGCCCCCAAAAGG
GCGAACGACAGCCTGGGGGGCCTCAGCCCCGCCCCACCCCCCTTCTTGTCTTCGCAGGGGCCCACCCCTC
TGCCCAGCTTCACTTCAGGGGTTCCCAGCCACGCGCCAGTCTCTGGTTCCCCCAGCACCCCACGCTCCTC
GGGCCCTGTGACAGCCACCAGCTCCCTGTACATCCCAGCCCCTAGTCGGCCTGTCACCCCAGGTGGAGCT
CCAGAGCCCCCCGCTCCTCCTAGCGCAGCTGCCATGACCTCCACCGCTTCTATCTTCCTATCTGCGCCTT
TGCGACCCTCTGCGCGCCCAGAGGCGCCTGCCCCAGGCCCAGGGGCTCCTGAGCCCCCCAGCGCTCGCGA
GCAGCGCATCTCTGTGCCAGCTGCCCGCACGGGTATCCTGCAGGAGGCCCGGCGCCGGGGGACCCGGAAG
CAGATGTTCCGGCCGGGAAAGGAGGAGACGAAGAACTCGCCCAACCCCGAGCTGCTATCGCTGGTACAGA
ACCTGGATGAAAAGCCTCGGGCCGGGGGTGCAGAATCTGGTCCTGAAGAAGATGCTCTGAGCCTCGGGGC
TGAAGCCTGCAACTTCATGCAGCCAGTAGGGGCCAGGAGTTACAAGACCCTGCCTCACGTGACACCTAAG
ACCCCCCCTCCAATGGCTCCCAAGACCCCGCCCCCTATGACTCCTAAGACTCCACCCCCAGTGGCTCCTA
AGCCCCCATCTCGAGGGCTCCTTGATGGGCTCGTGAATGGGGCAGCCTCTTCGGCTGGAATCCCTGAGCC
ACCAAGGCTGCAGGGCAGGGGTGGGGAGCTGTTTGCTAAGCGGCAGAGCCGTGCGGACAGGTATGTGGTG
GAAGGTACACCTGGTCCTGGTCTTGGCCCTCGGCCTAGAAGTCCTTCTCCTACCCCGTCTCTGCCCCCTT
CCTGGAAATATTCACCCAACATCCGTGCCCCGCCTCCTATTGCTTACAACCCACTGCTCTCTCCCTTTTT
CCCCCAGGCGGCCCGAACTCTCCCTAAGGCCCAATCCCAGGGGCCTCGGGCAACACCCAAGCAGGGCATC
AAGGCTCTAGATTTTATGCGGCATCAGCCCTATCAACTTAAAACTGCCATGTTCTGTTTTGATGAGGTTC
CCCCGACTCCTGGCCCTATCGCCTCAGGGTCCCCCAAAACTGCCCGAGTCCAGGAGATTCGCCGGTTTTC
CACTCCGGCACCCCAGCCCACTGCAGAACCCCTGGCTCCCACTGTGCTTGCCCCCCGAGCAGCCACTACA
CTGGATGAGCCCATCTGGAGAACAGAACTGGCCTCAGCCCCTGTTCCTAGCCCAGCCCCTCCTCCAGAGG
CTCCCAGGGGCCTTGGGGCTTCTCCCAGCTCCTGCGGTTTCCAGGTAGCCAGGCCCCGATTTTCAGCCAC
CAGAACAGGATTGCAAGCTCATGTGTGGAGGCCTGGGGCAGGGCACCAGTGAACAGGCACAGGTCCCAGG
ACCAAGGAGAGGTGGAACATCCAGTTCCTAAAGTTGCTTCTCCTACCCTATCCCATCCCCTGTCACGCAT
CTGGAAGCTAAATTGCCTCCTGCCAGAGATGGTTTCCAAGTTGATGTCCCCTTCCCCCACCTTCCTCCTC
ACTCTCTACCTCCCTGCCGCTTTCCAACCAAGTATGTCTGCTTTGGTATCTTTGCCTCTCTTTGTCTCTG
CATTTCCTTTCCTGGATCTCTGTCTTTATTTCCAGGCTTCTCCACCCATATTCCTCCACAGATCTCTCTT
CCTTGACATTTGTGCTTTTCTCCCTGGGCCTCATTTTAATGTTCAGTGAGAAGTAAACAGAGCAGAAGTG
ACCACTGGGACTTCAGGCAAGAAGCTCACCACCAGGCACACAGCAAAGGGACTGAACTGACCCCTGTTTG
CACTAAGCCACCCCCACCCCCCACTCTGCTTTCCCAAGCTTGACTGGCATATACCTAGGCCTGTGTGTGT
GTGTGTGTGTGTGTGTGTGTGTGTGTGTGCTCTTCCGTCTAAGGCATGAATAAGAGGGGAGGTCAAAA
TAAAGACCCAATCTGAGGCCGGGCACGGTGGCTCACGCCGGTAATCCCAGCACTTTGGAGGCCGAGGCG
GGCGGATCACGAGGTCAGGAGATCGAGACCATCCTGGCTAACACGGTGAAACCCCATTTCCACTAAAAAT
ACAAAAAATTAGCTGGGCGTGGTGGCGAGCGCCTGTAGTCCCAGCTACTCGGGAGGCTGAGGCAGGAGAA
TGGCATGAACCTGGAAGGCGGAGCTTGCAGTGAGCTGAGATTGCGCCACTGCACTCCAGCCTGGGCGACG

GAGCGAGACTCTGTCTCAAAACAAACAAACAAACAAAAGACCCAATCTGAGTCTTATCGTTGTACTGATA
GAAGGGTCAGATATCCCCACATGGAGTTGAGTGGGAGAAAGAGATTCACTAGAGAATAACTCCTTAGAGA
CCAATGTCTGTAGCAGGTGTACAGCATCTTGTGAAAGTTATGGAGCATGAAAAGACTGAAGGGCCAGGAC
AGTTTGCATGGGCTGAGTTATACCAGCTAGACCAGGAATAGAACAAAGAATTCTATACCTCAGGATTTCA
AAAAGTTAGCAACTTGAGAGGCCAGTGCTGAGCAACCCAGTACCCAGGAAATGAAAAAAAGAAAGAAAAT
TCCCTCCGAGAATGAACAAATCATTGGCTTCATTGCCTCATGAGCTTGAGAGAAAGGAGAAGAGAGCCAG
AGTGTGGCAAGTGAGGCCAAAATCAGAAGCATGGCAGAAATGAGTGTAAGTGATTGAGCCACAGACAGAA
GTGTGGCGAGGGACAATGCCATATTGGGAGAAGGTAAAGTTGAGTAACAAGAAACCAACCGTGTGTGAGA
GGGGGATTGGAAAAAAATTTGAGGGAGAAGAATGTTAGAATGGAAGGGAATGATGGTGGAAGGGAGGTGT
GAGGGTGTGTGCTGAGTGTTGAAAGAACGGTTGGTGTCTGTGTGATTTTCCTTGAGTCTGTTCTTCAGTG
TGTCTTCTGCAGCTTGCCATGACTGCCTGGGAAAGAGTAGGGAAATACCCAGAGCCAAAACCTCCTTTCA
GTCCCACCCCATCCCTCAAAACCCCAGCTATTGCTTCTTTTCAGCTTCAGGTCCTGATCTCCAATCTTAG
TATGGACTCCCTTCTCACCAAGACCACCACCAGCTACGTTTGCTGTGTAATCTGGAAAGTGATAATTTCC
TTTGCTTGTTGGGTGTGAGTCACAATACTTTGGTTTGTGCACAAGAATAAATTTATGCCCCATACCTTC

Inferred AA sequence from first ATG: METTPD

SYNPO2L.N2 cDNA
First exon highlighted in yellow, second exon highlighted in blue, ATG start codon highlighted in green.

CCTGGCTGGCAGTGGCTGCCTGAGCTCTCTACGTAGAGACAGTTCTGACCCCTGAAGAAGCCCACCCCAC
TACCATCCCCAGCTTGGGGTAAATAATATATTTAGCTGGCGACCTTGAGGTATCCTAAAGATTTGGAGTG
TTGCCTTGGAGAACAACACTCTAACTTTTCCTGCATCTTGGATGGAGACTACCCCTGACTGTGAGTAGCA
GGGGTGTGAAGCCCAGAGGACTGCCCTGGCAGTTGGAAGGCCGAGATCAACTTTAACTTCTGTCTAAACT
AACTGCAACGTGCAGAGAGCCTCCAAGAGAAGAGCATAAAAGAGGCCAAGACCAAATGCAGGACAATTGC
ATCCCTGCTCACTGCAGCCCCCAACCCCCACTCCAAAGGGGTACTTATGTTTAAGAAACGGCGGCAGAGA
GCCAAGAAGTACACCCTGGTGAGCTTCGGGGCTGCTGCTGGGACAGGCGCTGAGGAGGAGGACGGCGTTC
CCCCCACGAGTGAGTCCGAGCTGGACGAAGAAGCCTTCTCTGACGCCCGCAGCCTCACCAATCAATCTGA
CTGGGACAGTCCCTATCTGGACATGGAGCTTGCCAGGGCGGGCTCAAGAGCATCAGAGGGCCAGGGCTCT
GGGCTGGGAGGGCAGCTGAGTGAGGTCTCTGGGCGAGGGGTGCAGCTCTTTGAACAGCAGCGCCAGCGCG
CAGACTCCAGCACCCAGGAACTGGCACGGGTCGAACCAGCAGCCATGCTCAACGGGGAAGGCCTGCAGTC
ACCACCTCGGGCCCAGAGTGCTCCCCCAGAGGCAGCTGTGCTCCCACCCAGCCCCTTGCCGGCGCCTGTA
GCCAGCCCCAGACCCTTCCAACCAGGTGGTGGAGCCCCGACCCCAGCTCCAAGCATCTTTAACCGGTCAG
CCAGGCCCTTTACCCCGGGCCTACAAGGGCAGCGGCCAACTACCACCTCGGTTATTTTCCGGCCTTTAGC
CCCCAAAAGGGCGAACGACAGCCTGGGGGGCCTCAGCCCCGCCCCACCCCCCTTCTTGTCTTCGCAGGGG
CCCACCCCTCTGCCCAGCTTCACTTCAGGGGTTCCCAGCCACGCGCCAGTCTCTGGTTCCCCCAGCACCC
CACGCTCCTCGGGCCCTGTGACAGCCACCAGCTCCCTGTACATCCCAGCCCCTAGTCGGCCTGTCACCCC
AGGTGGAGCTCCAGAGCCCCCCGCTCCTCCTAGCGCAGCTGCCATGACCTCCACCGCTTCTATCTTCCTA
TCTGCGCCTTTGCGACCCTCTGCGCGCCCAGAGGCGCCTGCCCCAGGCCCAGGGGCTCCTGAGCCCCCCA
GCGCTCGCGAGCAGCGCATCTCTGTGCCAGCTGCCCGCACGGGTATCCTGCAGGAGGCCCGGCGCCGGGG
GACCCGGAAGCAGATGTTCCGGCCGGGAAAGGAGGAGACGAAGAACTCGCCCAACCCCGAGCTGCTATCG
CTGGTACAGAACCTGGATGAAAAGCCTCGGGCCGGGGGTGCAGAATCTGGTCCTGAAGAAGATGCTCTGA
GCCTCGGGGCTGAAGCCTGCAACTTCATGCAGCCAGTAGGGGCCAGGAGTTACAAGACCCTGCCTCACGT
GACACCTAAGACCCCCCCTCCAATGGCTCCCAAGACCCCGCCCCCTATGACTCCTAAGACTCCACCCCCA
GTGGCTCCTAAGCCCCCATCTCGAGGGCTCCTTGATGGGCTCGTGAATGGGGCAGCCTCTTCGGCTGGAA
TCCCTGAGCCACCAAGGCTGCAGGGCAGGGGTGGGGAGCTGTTTGCTAAGCGGCAGAGCCGTGCGGACAG
GTATGTGGTGGAAGGTACACCTGGTCCTGGTCTTGGCCCTCGGCCTAGAAGTCCTTCTCCTACCCCGTCT
CTGCCCCCTTCCTGGAAATATTCACCCAACATCCGTGCCCCGCCTCCTATTGCTTACAACCCACTGCTCT
CTCCCTTTTTCCCCCAGGCGGCCCGAACTCTCCCTAAGGCCCAATCCCAGGGGCCTCGGGCAACACCCAA

GCAGGGCATCAAGGCTCTAGATTTTATGCGGCATCAGCCCTATCAACTTAAAACTGCCATGTTCTGTTTT
GATGAGGTTCCCCCGACTCCTGGCCCTATCGCCTCAGGGTCCCCCAAAACTGCCCGAGTCCAGGAGATTC
GCCGGTTTTCCACTCCGGCACCCCAGCCCACTGCAGAACCCCTGGCTCCCACTGTGCTTGCCCCCCGAGC
AGCCACTACACTGGATGAGCCCATCTGGAGAACAGAACTGGCCTCAGCCCCTGTTCCTAGCCCAGCCCCT
CCTCCAGAGGCTCCCAGGGGCCTTGGGGCTTCTCCCAGCTCCTGCGGTTTCCAGGTAGCCAGGCCCCGAT
TTTCAGCCACCAGAACAGGATTGCAAGCTCATGTGTGGAGGCCTGGGGCAGGGCACCAGTGAACAGGCAC
AGGTCCCAGGACCAAGGAGAGGTGGAACATCCAGTTCCAAAGTTGCTTCTCCTACCCTATCCCATCCCCT
GTCACGCATCTGGAAGCTAAATTGCCTCCTGCCAGAGATGGTTTCCAAGTTGATGTCCCCTTCCCCCACC
TTCCTCCTCACTCTCTACCTCCCTGCCGCTTTCCAACCAAGTATGTCTGCTTTGGTATCTTTGCCTCTCT
TTGTCTCTGCATTTCCTTTCCTGGATCTCTGTCTTTATTTCCAGGCTTCTCCACCCATATTCCTCCACAG
ATCTCTCTTCCTTGACATTTGTGCTTTTCTCCCTGGGCCTCATTTTAATGTTCAGTGAGAAGTAAACAGA
GCAGAAGTGACCACTGGGACTTCAGGCAAGAAGCTCACCACCAGGCACACAGCAAAGGGACTGAACTGAC
CCCTGTTTGCACTAAGCCACCCCCACCCCCCACTCTGCTTTCCCAAGCTTGACTGGCATATACCTAGGCC
TGTGTGTGTGTGTGTGTGTGTGTGTGTGTGTGTGTGTGTGTGCTCTTCCGTCTAAGGCATGAATAAGAGGGG
AGGTCAAAATAAAGACCCAATCTGAGGCCGGGCACGGTGGCTCACGCCGGTAATCCCAGCACTTTGGGAG
GCCGAGGCGGGCGGATCACGAGGTCAGGAGATCGAGACCATCCTGGCTAACACGGTGAAACCCCATTTCC
ACTAAAAATACAAAAAATTAGCTGGGCGTGGTGGCGAGCGCCTGTAGTCCCAGCTACTCGGGAGGCTGAG
GCAGGAGAATGGCATGAACCTGGAAGGCGGAGCTTGCAGTGAGCTGAGATTGCGCCACTGCACTCCAGCC
TGGGCGACGGAGCGAGACTCTGTCTCAAAACAAACAAACAAACAAAAGACCCAATCTGAGTCTTATCGTT
GTACTGATAGAAGGGTCAGATATCCCCACATGGAGTTGAGTGGGAGAAAGAGATTCACTAGAGAATAACT
CCTTAGAGACCAATGTCTGTAGCAGGTGTACAGCATCTTGTGAAAGTTATGGAGCATGAAAAGACTGAAG
GGCCAGGACAGTTTGCATGGGCTGAGTTATACCAGCTAGACCAGGAATAGAACAAAGAATTCTATACCTC
AGGATTTCAAAAAGTTAGCAACTTGAGAGGCCAGTGCTGAGCAACCCAGTACCCAGGAAATGAAAAAAAG
AAAGAAAATTCCCTCCGAGAATGAACAAATCATTGGCTTCATTGCCTCATGAGCTTGAGAGAAAGGAGAA
GAGAGCCAGAGTGTGGCAAGTGAGGCCAAAATCAGAAGCATGGCAGAAATGAGTGTAAGTGATTGAGCCA
CAGACAGAAGTGTGGCGAGGGACAATGCCATATTGGGAGAAGGTAAAGTTGAGTAACAAGAAACCAACCG
TGTGTGAGAGGGGGATTGGAAAAAAATTTGAGGGAGAAGAATGTTAGAATGGAAGGGAATGATGGTGGAA
GGGAGGTGTGAGGGTGTGTGCTGAGTGTTGAAAGAACGGTTGGTGTCTGTGTGATTTTCCTTGAGTCTGT
TCTTCAGTGTGTCTTCTGCAGCTTGCCATGACTGCCTGGGAAAGAGTAGGGAAATACCCAGAGCCAAAAC
CTCCTTTCAGTCCCACCCCATCCCTCAAAACCCCAGCTATTGCTTCTTTTCAGCTTCAGGTCCTGATCTC
CAATCTTAGTATGGACTCCCTTCTCACCAAGACCACCACCAGCTACGTTTGCTGTGTAATCTGGAAAGTG
ATAATTTCCTTTGCTTGTTGGGTGTGAGTCACAATACTTTGGTTTGTGCACAAGAATAAATTTATGCCCC
ATACCTTC

Inferred AA sequence from first ATG: METTPDCE

Supplemental File S2. Annotations of all SNPs in high LD with rs60212594 (r^2>0.8)

SNP	new SNP	LD (r2)	Distance	H3K4 me1	H3K4 me3	H3K9 ac	DNase1 HS	MYOZ-1 Euro descent eQTL beta	MYOZ-1 Euro descent p-value (-log10)	SYNPO2L Euro descent eQTL beta	SYNPO2L Euro descent p-value (-log10)
rs60212594		1	0	Yes	Yes	Yes	Yes	2.02	44.25	-0.23	13.43
rs60632610		0.9833	1333	Yes	Yes	Yes	Yes	1.99	42.09	-0.23	13.2
rs11000728		0.9666	-10044	Yes	Yes	Yes	Yes	2.05	42.64	-0.24	13.04
rs34163229		0.9666	-7432	Yes	Yes	Yes	Yes	2.05	43.81	-0.24	13.14
rs3740293		0.9666	-8203	Yes	Yes	Yes	Yes	2.06	43.79	-0.24	13.09
rs3812629		0.9666	-7054	Yes	Yes	Yes	Yes	2.05	43.81	-0.24	13.13
rs41280404		0.9666	-8709	Yes	Yes	Yes		2.05	42.65	-0.24	13.05
rs4746139		0.9666	-6695	Yes	Yes	Yes		2.05	43.82	-0.24	13.13
rs7900932		0.9511	6226					1.93	34.26	-0.21	10.14
rs7915134		0.9511	5836					1.94	39.48	-0.22	12.34
rs58165775	rs7394178	0.9433	7236					1.94	39.45	-0.22	12.34
rs61057415	rs7394152	0.9433	7102					1.94	39.45	-0.22	12.34
rs78249997		0.935	7970					1.94	39.18	-0.22	12.43
rs148321568		0.9266	13952					1.95	36.56	-0.23	12.01
rs4745721		0.877	50243					1.94	32.99	-0.21	9.24
rs76192127		0.877	48166					1.95	33.33	-0.21	9.24
rs2177843		0.8766	-4467	Yes	Yes	Yes	Yes	1.99	42.09	-0.23	13.04
rs3878005		0.8694	16734					2	38.17	-0.22	11.32
rs76522838		0.8694	36557					N.A			
rs147790633		0.8619	33238					1.94	39.12	-0.22	11.36
rs76443711		0.8619	35445					1.97	39.29	-0.22	11.47

rs11000752		0.8531	56011					1.89	36.62	-0.21	11.22
rs11000764		0.8531	92520					1.88	35.9	-0.21	11.1
rs11000766		0.8531	98608					1.86	35.27	-0.21	11.02
rs11000771		0.8531	110114					1.86	35.2	-0.21	10.94
rs11000772		0.8531	119644	Yes	Yes	Yes		1.86	35.18	-0.21	11
rs11000773		0.8531	119771	Yes	Yes	Yes	Yes	1.86	35.18	-0.21	11
rs11000777		0.8531	130719	Yes	Yes	Yes	Yes	1.86	35.25	-0.21	10.63
rs11541237		0.8531	116618	Yes	Yes	Yes		1.86	35.18	-0.21	11
rs117934466	rs3088070	0.8531	115669					1.86	35.18	-0.21	11
rs1589541826		0.8531	127216				Yes				
rs2292307		0.8531	135572					1.86	35.18	-0.21	11
rs2306325		0.8531	92330					1.88	35.92	-0.21	11.1
rs4082339		0.8531	125468	Yes	Yes			1.86	35.18	-0.21	11
rs4746145		0.8531	103643					1.86	35.22	-0.21	11.01
rs4746146		0.8531	112853					1.86	35.18	-0.21	11
rs4746147		0.8531	113364					1.84	34.74	-0.21	11.19
rs58747017	rs3781216	0.8531	108060					1.86	35.18	-0.21	11
rs60035714	rs7099599	0.8531	72737					1.88	36.14	-0.21	11.13
rs60809795		0.8531	109677					1.86	35.18	-0.21	11
rs76033784		0.8531	121626	Yes		Yes		1.86	35.18	-0.21	11
rs78790589		0.8531	125254	Yes				1.86	35.18	-0.21	11
rs78845904		0.8531	102905					1.86	35.23	-0.21	11.01
rs78985317		0.8531	100121					1.86	35.26	-0.21	11.01
rs79936644		0.8531	130773	Yes	Yes	Yes	Yes	1.86	35.25	-0.21	10.63

rs11000775		0.8456	124666	Yes				1.86	35.18	-0.21	11
rs11000780		0.8456	144733					1.86	35.2	-0.21	11.03
rs1174183105		0.8456	97542					N.A.			
rs12217245		0.8456	162139	Yes		Yes		1.84	34.81	-0.21	11.22
rs12220394		0.8456	162061	Yes		Yes		1.84	34.81	-0.21	11.22
rs16930909		0.8456	147238					1.85	35.05	-0.21	11.19
rs2075641		0.8456	147764					1.85	34.97	-0.21	11.2
rs57746267	rs2306327	0.8456	159434					1.84	34.81	-0.21	11.21
rs60428456	rs4114992	0.8456	152485					1.84	34.8	-0.21	11.21
rs11000774		0.8449	124499	Yes				N.A.			
rs188726810		0.8382	165882					1.75	33.13	-0.2	10.83
rs59693993		0.8382	168690					1.84	34.73	-0.21	11.26
rs3198002	rs1057410	0.8374	159735					1.84	34.81	-0.21	11.21
rs4746151		0.8374	164376	Yes				1.85	34.74	-0.21	11.26
rs59204697	rs3843939	0.8374	164604					1.85	34.74	-0.21	11.26
rs12572278		0.8368	90169		Yes	Yes	Yes	1.88	35.66	-0.21	11.2
rs57727673	rs12573448	0.8368	90168		Yes	Yes	Yes	1.88	35.66	-0.21	11.2
rs11000734		0.8324	5319					1.87	37.54	-0.21	11.92
rs61235197	rs125701126	0.8324	2445					N.A.			
rs1035227203		0.8309	154415					N.A.			
rs4746140		0.8262	2905	Yes		Yes		1.86	37.54	-0.21	11.91
rs6480708		0.8262	5770					1.87	37.54	-0.21	11.92
rs10824026		0.8201	6864					1.87	37.54	-0.21	11.92

Supplemental File S3. MYOZ1 coexpression, unadjusted P-value < 0.001

ensembl.gene.id	hgid	description	P.Value	partialR
ENSG00000177791	MYOZ1	myozenin 1 [Source:HGNC Symbol;Acc:13752]	0	0.9999506
ENSG00000166317	SYNPO2L	synaptopodin 2-like [Source:HGNC Symbol;Acc:23532]	3.61E-15	-0.517342
ENSG00000196968	FUT11	fucosyltransferase 11 (alpha (1,3) fucosyltransferase) [Source:HGNC Symbol;Acc:19233]	6.87E-06	-0.311198
ENSG00000166321	NUDT13	nudix (nucleoside diphosphate linked moiety X)-type motif 13 [Source:HGNC Symbol;Acc:18827]	2.26E-05	-0.294057
ENSG00000007047	MARK4	MAP/microtubule affinity-regulating kinase 4 [Source:HGNC Symbol;Acc:13538]	8.88E-05	0.2728427
ENSG00000063761	ADCK1	aarF domain containing kinase 1 [Source:HGNC Symbol;Acc:19038]	0.000133	-0.266256
ENSG00000174175	SELP	selectin P (granule membrane protein 140kDa, antigen CD62) [Source:HGNC Symbol;Acc:10721]	0.000134	-0.266143
ENSG00000261620	AC106785.4	high mobility group nucleosomal binding domain 2 pseudogene 41 [Source:HGNC Symbol;Acc:39412]	0.000147	0.2645318
ENSG00000229570	GAPDHP58	glyceraldehyde 3 phosphate dehydrogenase pseudogene 58 [Source:HGNC Symbol;Acc:4142]	0.000184	0.260805
ENSG00000102010	BMX	BMX non-receptor tyrosine kinase [Source:HGNC Symbol;Acc:1079]	0.000208	-0.258732
ENSG00000147697	GSDMC	gasdermin C [Source:HGNC Symbol;Acc:7151]	0.000218	-0.257922
ENSG00000182858	ALG12	ALG12, alpha-1,6-mannosyltransferase [Source:HGNC Symbol;Acc:19358]	0.00022	-0.257784
ENSG00000196588	MKL1	megakaryoblastic leukemia (translocation) 1 [Source:HGNC Symbol;Acc:14334]	0.000234	-0.256711
ENSG00000257264	AC136443.3	microRNA 3179-1 [Source:HGNC Symbol;Acc:38353]	0.000308	-0.251927
ENSG00000118689	FOXO3	forkhead box O3 [Source:HGNC Symbol;Acc:3821]	0.00037	-0.248724
ENSG00000256948	AC026369.3		0.000413	0.2467357
ENSG00000229048	DUTP1	deoxyuridine triphosphatase pseudogene 1 [Source:HGNC Symbol;Acc:31956]	0.000436	0.2457614
ENSG00000103241	FOXF1	forkhead box F1 [Source:HGNC Symbol;Acc:3809]	0.000459	0.2448497
ENSG00000168490	PHYHIP	phytanoyl-CoA 2-hydroxylase interacting protein [Source:HGNC Symbol;Acc:16865]	0.000499	0.2433479
ENSG00000146409	SLC18B1	solute carrier family 18, subfamily B, member 1 [Source:HGNC Symbol;Acc:21573]	0.000503	0.2432114
ENSG00000230552	AC092162.2		0.000513	-0.242848
ENSG00000161618	ALDH16A1	aldehyde dehydrogenase 16 family, member A1 [Source:HGNC Symbol;Acc:28114]	0.000527	-0.242331
ENSG00000113441	LNPEP	leucyl/cystinyl aminopeptidase [Source:HGNC Symbol;Acc:6656]	0.000547	0.2416621
ENSG00000124762	CDKN1A	cyclin-dependent kinase inhibitor 1A (p21, Cip1) [Source:HGNC Symbol;Acc:1784]	0.000585	0.240436
ENSG00000233163	RPS12P17		0.000592	0.2402057
ENSG00000170456	DENND5B	DENN/MADD domain containing 5B [Source:HGNC Symbol;Acc:28338]	0.000634	0.2389394
ENSG00000180089	TMEM86B	transmembrane protein 86B [Source:HGNC Symbol;Acc:28448]	0.000646	-0.23861
ENSG00000261635	AC103988.2		0.00066	-0.238219
ENSG00000166348	USP54	ubiquitin specific peptidase 54 [Source:HGNC Symbol;Acc:23513]	0.000672	0.237885
ENSG00000250030	AC104806.1		0.000687	-0.237474
ENSG00000267865			0.000713	0.2367792
ENSG00000259787			0.000713	0.2367704
ENSG00000185669	SNAI3	snail family zinc finger 3 [Source:HGNC Symbol;Acc:18411]	0.000717	0.2366802
ENSG00000228075	BOD1L2	biorientation of chromosomes in cell division 1-like 2 [Source:HGNC Symbol;Acc:28505]	0.000761	0.2355617

ENSG00000184164	CRELD2	cysteine-rich with EGF-like domains 2 [Source:HGNC Symbol;Acc:28150]	0.000817	-0.234221
ENSG00000223486	AC092198.1		0.000823	-0.234077
ENSG00000250500	AC017007.4		0.000828	-0.233966
ENSG00000172380	GNG12	guanine nucleotide binding protein (G protein), gamma 12 [Source:HGNC Symbol;Acc:19663]	0.000872	-0.232978
ENSG00000152670	DDX4	DEAD (Asp-Glu-Ala-Asp) box polypeptide 4 [Source:HGNC Symbol;Acc:18700]	0.000901	-0.23237
ENSG00000178445	GLDC	glycine dehydrogenase (decarboxylating) [Source:HGNC Symbol;Acc:4313]	0.000904	-0.232305
ENSG00000159167	STC1	stanniocalcin 1 [Source:HGNC Symbol;Acc:11373]	0.000926	0.2318565
ENSG00000253844	AC064807.2		0.000949	0.2313741
ENSG00000253766	AC091946.1		0.00095	0.2313615
ENSG00000181035	SLC25A42	solute carrier family 25, member 42 [Source:HGNC Symbol;Acc:28380]	0.000954	0.2312754
ENSG00000259344			0.000982	0.2307194

Supplemental File S4. Coexpression with SYNPO2L, unadjusted P-value < 0.001

ensembl.gene.id	hgid	description	P.Value	partialR
ENSG00000166317	SYNPO2L	synaptopodin 2-like [Source:HGNC Symbol;Acc:23532]	3.12E-246	0.998227
ENSG00000177791	MYOZ1	myozenin 1 [Source:HGNC Symbol;Acc:13752]	8.90E-14	-0.495119
ENSG00000136153	LMO7	LIM domain 7 [Source:HGNC Symbol;Acc:6646]	1.54E-12	0.472715
ENSG00000203867	RBM20	RNA binding motif protein 20 [Source:HGNC Symbol;Acc:27424]	1.31E-10	0.434222
ENSG00000157036	EXOG	endo/exonuclease (5'-3'), endonuclease G-like [Source:HGNC Symbol;Acc:3347]	1.55E-10	0.432653
ENSG00000047849	MAP4	microtubule-associated protein 4 [Source:HGNC Symbol;Acc:6862]	7.68E-10	0.417407
ENSG00000162614	NEXN	nexilin (F actin binding protein) [Source:HGNC Symbol;Acc:29557]	8.16E-10	0.416815
ENSG00000172399	MYOZ2	myozenin 2 [Source:HGNC Symbol;Acc:1330]	1.09E-09	0.414013
ENSG00000149596	JPH2	junctophilin 2 [Source:HGNC Symbol;Acc:14202]	1.77E-09	0.409164
ENSG00000168509	HFE2	hemochromatosis type 2 (juvenile) [Source:HGNC Symbol;Acc:4887]	3.28E-09	0.402927
ENSG00000165410	CFL2	cofilin 2 (muscle) [Source:HGNC Symbol;Acc:1875]	4.76E-09	0.399063
ENSG00000101605	MYOM1	myomesin 1 [Source:HGNC Symbol;Acc:7613]	4.84E-09	0.398902
ENSG00000116688	MFN2	mitofusin 2 [Source:HGNC Symbol;Acc:16877]	1.87E-08	0.384565
ENSG00000150281	CTF1	cardiotrophin 1 [Source:HGNC Symbol;Acc:2499]	1.87E-08	0.384564
ENSG00000101413	RPRD1B	regulation of nuclear pre-mRNA domain containing 1B [Source:HGNC Symbol;Acc:16209]	2.03E-08	0.383672
ENSG00000167987	VPS37C	vacuolar protein sorting 37 homolog C (S. cerevisiae) [Source:HGNC Symbol;Acc:26097]	2.53E-08	0.381223
ENSG00000187778	MCRS1	microspherule protein 1 [Source:HGNC Symbol;Acc:6960]	3.40E-08	0.377962
ENSG00000122986	HVCN1	hydrogen voltage-gated channel 1 [Source:HGNC Symbol;Acc:28240]	4.77E-08	-0.374174
ENSG00000113578	FGF1	fibroblast growth factor 1 (acidic) [Source:HGNC Symbol;Acc:3665]	6.72E-08	0.370298
ENSG00000125741	OPA3	optic atrophy 3 (autosomal recessive, with chorea and spastic paraplegia) [Source:HGNC Symbol;Acc:8142]	8.96E-08	0.366987
ENSG00000165152	TMEM246	transmembrane protein 246 [Source:HGNC Symbol;Acc:28180]	9.90E-08	0.365834
ENSG00000122367	LDB3	LIM domain binding 3 [Source:HGNC Symbol;Acc:15710]	1.02E-07	0.365527
ENSG00000073711	PPP2R3A	protein phosphatase 2, regulatory subunit B'', alpha [Source:HGNC Symbol;Acc:9307]	1.21E-07	0.363451
ENSG00000122477	LRRC39	leucine rich repeat containing 39 [Source:HGNC Symbol;Acc:28228]	1.29E-07	0.36276
ENSG00000132688	NES	nestin [Source:HGNC Symbol;Acc:7756]	1.85E-07	0.358473
ENSG00000074590	NUAK1	NUAK family, SNF1-like kinase, 1 [Source:HGNC Symbol;Acc:14311]	2.32E-07	0.35582
ENSG00000095637	SORBS1	sorbin and SH3 domain containing 1 [Source:HGNC Symbol;Acc:14565]	2.91E-07	0.353054
ENSG00000170681	CAVIN4	muscle-related coiled-coil protein [Source:HGNC Symbol;Acc:33742]	3.34E-07	0.351392
ENSG00000058056	USP13	ubiquitin specific peptidase 13 (isopeptidase T-3) [Source:HGNC Symbol;Acc:12611]	4.61E-07	0.347441
ENSG00000023228	NDUFS1	NADH dehydrogenase (ubiquinone) Fe-S protein 1, 75kDa (NADH-coenzyme Q reductase) [Source:HGNC Symbol;Acc:77(	5.77E-07	0.344641
ENSG00000101608	MYL12A	myosin, light chain 12A, regulatory, non-sarcomeric [Source:HGNC Symbol;Acc:16701]	6.53E-07	0.343119
ENSG00000165887	ANKRD2	ankyrin repeat domain 2 (stretch responsive muscle) [Source:HGNC Symbol;Acc:495]	7.37E-07	0.341598
ENSG00000134571	MYBPC3	myosin binding protein C, cardiac [Source:HGNC Symbol;Acc:7551]	8.40E-07	0.33993
ENSG00000119650	IFT43	intraflagellar transport 43 homolog (Chlamydomonas) [Source:HGNC Symbol;Acc:29669]	8.74E-07	0.339431

ENSG00000160460	SPTBN4	spectrin, beta, non-erythrocytic 4 [Source:HGNC Symbol;Acc:14896]	1.25E-06	-0.334847
ENSG00000057294	PKP2	plakophilin 2 [Source:HGNC Symbol;Acc:9024]	1.67E-06	0.331083
ENSG00000111652	COPS7A	COP9 signalosome subunit 7A [Source:HGNC Symbol;Acc:16758]	1.89E-06	0.329472
ENSG00000168427	KLHL30	kelch-like family member 30 [Source:HGNC Symbol;Acc:24770]	1.92E-06	0.329243
ENSG00000175662	TOM1L2	target of myb1-like 2 (chicken) [Source:HGNC Symbol;Acc:11984]	2.17E-06	0.327654
ENSG00000185739	SRL	sarcalumenin [Source:HGNC Symbol;Acc:11295]	2.42E-06	0.326209
ENSG00000159166	LAD1	ladinin 1 [Source:HGNC Symbol;Acc:6472]	2.45E-06	0.326051
ENSG00000077522	ACTN2	actinin, alpha 2 [Source:HGNC Symbol;Acc:164]	2.91E-06	0.323738
ENSG00000196204	RNF216P1	ring finger protein 216 pseudogene 1 [Source:HGNC Symbol;Acc:33610]	3.58E-06	0.320935
ENSG00000173402	DAG1	dystroglycan 1 (dystrophin-associated glycoprotein 1) [Source:HGNC Symbol;Acc:2666]	3.58E-06	0.320914
ENSG00000091482	SMPX	small muscle protein, X-linked [Source:HGNC Symbol;Acc:11122]	4.04E-06	0.3193
ENSG00000185761	ADAMTSL5	ADAMTS-like 5 [Source:HGNC Symbol;Acc:27912]	4.06E-06	0.319214
ENSG00000163681	SLMAP	sarcolemma associated protein [Source:HGNC Symbol;Acc:16643]	4.12E-06	0.319008
ENSG00000158882	TOMM40L	translocase of outer mitochondrial membrane 40 homolog (yeast)-like [Source:HGNC Symbol;Acc:25756]	4.36E-06	0.318239
ENSG00000265399	AP005329.2		4.37E-06	0.31824
ENSG00000186628	FSD2	fibronectin type III and SPRY domain containing 2 [Source:HGNC Symbol;Acc:18024]	6.16E-06	0.313487
ENSG00000126773	PCNX4	pecanex-like 4 (Drosophila) [Source:HGNC Symbol;Acc:20349]	6.71E-06	0.312284
ENSG00000188549	C15orf52	chromosome 15 open reading frame 52 [Source:HGNC Symbol;Acc:33488]	7.32E-06	0.311052
ENSG00000261667	LY6L	Uncharacterized protein [Source:UniProtKB/TrEMBL;Acc:H3BQJ8]	7.78E-06	-0.310202
ENSG00000170759	KIF5B	kinesin family member 5B [Source:HGNC Symbol;Acc:6324]	8.23E-06	0.309415
ENSG00000186298	PPP1CC	protein phosphatase 1, catalytic subunit, gamma isozyme [Source:HGNC Symbol;Acc:9283]	8.26E-06	-0.309356
ENSG00000114867	EIF4G1	eukaryotic translation initiation factor 4 gamma, 1 [Source:HGNC Symbol;Acc:3296]	8.87E-06	0.308355
ENSG00000182533	CAV3	caveolin 3 [Source:HGNC Symbol;Acc:1529]	9.09E-06	0.308019
ENSG00000129474	AJUBA	ajuba LIM protein [Source:HGNC Symbol;Acc:20250]	9.12E-06	0.307966
ENSG00000088970	KIZ	polo-like kinase 1 substrate 1 [Source:HGNC Symbol;Acc:15865]	9.48E-06	-0.307402
ENSG00000179564	LSMEM2	leucine-rich single-pass membrane protein 2 [Source:HGNC Symbol;Acc:26781]	9.79E-06	0.306955
ENSG00000198523	PLN	phospholamban [Source:HGNC Symbol;Acc:9080]	9.89E-06	0.306798
ENSG00000101940	WDR13	WD repeat domain 13 [Source:HGNC Symbol;Acc:14352]	1.01E-05	0.306442
ENSG00000236301	MRGPRG-AS	MRGPRG antisense RNA 1 [Source:HGNC Symbol;Acc:26691]	1.02E-05	-0.306387
ENSG00000164068	RNF123	ring finger protein 123 [Source:HGNC Symbol;Acc:21148]	1.04E-05	0.306056
ENSG00000226733	AL138826.1		1.13E-05	-0.304888
ENSG00000157881	PANK4	pantothenate kinase 4 [Source:HGNC Symbol;Acc:19366]	1.15E-05	0.304702
ENSG00000170190	SLC16A5	solute carrier family 16 (monocarboxylate transporter), member 5 [Source:HGNC Symbol;Acc:10926]	1.21E-05	0.303863
ENSG00000010256	UQCRC1	ubiquinol-cytochrome c reductase core protein I [Source:HGNC Symbol;Acc:12585]	1.22E-05	0.303738
ENSG00000166930	MS4A5	membrane-spanning 4-domains, subfamily A, member 5 [Source:HGNC Symbol;Acc:13374]	1.32E-05	-0.302642
ENSG00000186967	KRTAP19-4	keratin associated protein 19-4 [Source:HGNC Symbol;Acc:18939]	1.32E-05	-0.302642

ENSG00000187170	LCE4A	late cornified envelope 4A [Source:HGNC Symbol;Acc:16613]	1.32E-05	-0.302642
ENSG00000201102	Y_RNA	Y RNA [Source:RFAM;Acc:RF00019]	1.32E-05	-0.302642
ENSG00000213247	AL355863.1		1.32E-05	-0.302642
ENSG00000220831	NDUFA5P9	NADH dehydrogenase (ubiquinone) 1 alpha subcomplex, 5 pseudogene 9 [Source:HGNC Symbol;Acc:48851]	1.32E-05	-0.302642
ENSG00000223780		meprin A, alpha pseudogene 2 [Source:HGNC Symbol;Acc:7017]	1.32E-05	-0.302642
ENSG00000224519	HMGN1P19	high mobility group nucleosome binding domain 1 pseudogene 19 [Source:HGNC Symbol;Acc:39363]	1.32E-05	-0.302642
ENSG00000225144	AC018643.1		1.32E-05	-0.302642
ENSG00000227592	PIGFP3	phosphatidylinositol glycan anchor biosynthesis, class F, pseudogene 3 [Source:HGNC Symbol;Acc:45151]	1.32E-05	-0.302642
ENSG00000235667			1.32E-05	-0.302642
ENSG00000243583			1.32E-05	-0.302642
ENSG00000249722	AC025752.1		1.32E-05	-0.302642
ENSG00000251588	AC125336.1		1.32E-05	-0.302642
ENSG00000255183	AP000756.1		1.32E-05	-0.302642
ENSG00000255730	AC011462.1	2-oxoisovalerate dehydrogenase subunit alpha, mitochondrial; Uncharacterized protein [Source:UniProtKB/TrEMBL;Acc	1.32E-05	-0.302642
ENSG00000258223	PRSS58	protease, serine, 58 [Source:HGNC Symbol;Acc:39125]	1.32E-05	-0.302642
ENSG00000258346	AC079384.1		1.32E-05	-0.302642
ENSG00000258874	AC009396.3		1.32E-05	-0.302642
ENSG00000221890	NPTXR	neuronal pentraxin receptor [Source:HGNC Symbol;Acc:7954]	1.36E-05	0.302214
ENSG00000090432	MUL1	mitochondrial E3 ubiquitin protein ligase 1 [Source:HGNC Symbol;Acc:25762]	1.43E-05	0.30145
ENSG00000143420	ENSA	endosulfine alpha [Source:HGNC Symbol;Acc:3360]	1.45E-05	0.301323
ENSG00000161558	TMEM143	transmembrane protein 143 [Source:HGNC Symbol;Acc:25603]	1.45E-05	0.301269
ENSG00000266153	AP005380.1		1.48E-05	-0.300939
ENSG00000127824	TUBA4A	tubulin, alpha 4a [Source:HGNC Symbol;Acc:12407]	1.51E-05	0.300708
ENSG00000035403	VCL	vinculin [Source:HGNC Symbol;Acc:12665]	1.51E-05	0.300676
ENSG00000165912	PACSIN3	protein kinase C and casein kinase substrate in neurons 3 [Source:HGNC Symbol;Acc:8572]	1.56E-05	0.300182
ENSG00000154556	SORBS2	sorbin and SH3 domain containing 2 [Source:HGNC Symbol;Acc:24098]	2.00E-05	0.296596
ENSG00000162409	PRKAA2	protein kinase, AMP-activated, alpha 2 catalytic subunit [Source:HGNC Symbol;Acc:9377]	2.00E-05	0.29658
ENSG00000119383	PTPA	protein phosphatase 2A activator, regulatory subunit 4 [Source:HGNC Symbol;Acc:9308]	2.01E-05	0.29651
ENSG00000107165	TYRP1	tyrosinase-related protein 1 [Source:HGNC Symbol;Acc:12450]	2.10E-05	0.295873
ENSG00000085998	POMGNT1	protein O-linked mannose N-acetylglucosaminyltransferase 1 (beta 1,2-) [Source:HGNC Symbol;Acc:19139]	2.13E-05	0.295648
ENSG00000186591	UBE2H	ubiquitin-conjugating enzyme E2H [Source:HGNC Symbol;Acc:12484]	2.25E-05	0.294812
ENSG00000134765	DSC1	desmocollin 1 [Source:HGNC Symbol;Acc:3035]	2.43E-05	0.29365
ENSG00000183454	GRIN2A	glutamate receptor, ionotropic, N-methyl D-aspartate 2A [Source:HGNC Symbol;Acc:4585]	2.57E-05	0.292831
ENSG00000250303	AP002884.3		2.88E-05	0.291137
ENSG00000152413	HOMER1	homer homolog 1 (Drosophila) [Source:HGNC Symbol;Acc:17512]	2.97E-05	0.290658
ENSG00000156463	SH3RF2	SH3 domain containing ring finger 2 [Source:HGNC Symbol;Acc:26299]	3.29E-05	0.289097

ENSG00000135916	ITM2C	integral membrane protein 2C [Source:HGNC Symbol;Acc:6175]	3.31E-05	0.28901
ENSG00000130881	LRP3	low density lipoprotein receptor-related protein 3 [Source:HGNC Symbol;Acc:6695]	3.46E-05	-0.288296
ENSG00000176087	SLC35A4	solute carrier family 35, member A4 [Source:HGNC Symbol;Acc:20753]	3.59E-05	0.287765
ENSG00000063761	ADCK1	aarF domain containing kinase 1 [Source:HGNC Symbol;Acc:19038]	3.60E-05	0.28769
ENSG00000177354	C10orf71	chromosome 10 open reading frame 71 [Source:HGNC Symbol;Acc:26973]	4.00E-05	0.286078
ENSG00000135540	NHSL1	NHS-like 1 [Source:HGNC Symbol;Acc:21021]	4.08E-05	0.285783
ENSG00000163491	NEK10	NIMA-related kinase 10 [Source:HGNC Symbol;Acc:18592]	4.24E-05	0.285201
ENSG00000092054	MYH7	myosin, heavy chain 7, cardiac muscle, beta [Source:HGNC Symbol;Acc:7577]	4.29E-05	0.285013
ENSG00000250959	GLUD1P3	glutamate dehydrogenase 1 pseudogene 3 [Source:HGNC Symbol;Acc:4338]	4.34E-05	-0.284833
ENSG00000169570	DTWD2	DTW domain containing 2 [Source:HGNC Symbol;Acc:19334]	4.71E-05	0.283564
ENSG00000175084	DES	desmin [Source:HGNC Symbol;Acc:2770]	4.72E-05	0.283542
ENSG00000259721	AC090877.2		4.76E-05	-0.283385
ENSG00000002330	BAD	BCL2-associated agonist of cell death [Source:HGNC Symbol;Acc:936]	4.93E-05	-0.282848
ENSG00000137076	TLN1	talin 1 [Source:HGNC Symbol;Acc:11845]	5.06E-05	0.282432
ENSG00000134716	CYP2J2	cytochrome P450, family 2, subfamily J, polypeptide 2 [Source:HGNC Symbol;Acc:2634]	5.16E-05	0.282154
ENSG00000127948	POR	P450 (cytochrome) oxidoreductase [Source:HGNC Symbol;Acc:9208]	5.17E-05	-0.282092
ENSG00000100982	PCIF1	PDX1 C-terminal inhibiting factor 1 [Source:HGNC Symbol;Acc:16200]	5.27E-05	0.281814
ENSG00000020129	NCDN	neurochondrin [Source:HGNC Symbol;Acc:17597]	5.29E-05	0.281744
ENSG00000231837	RPS7P2	ribosomal protein S7 pseudogene 2 [Source:HGNC Symbol;Acc:36079]	5.43E-05	-0.281335
ENSG00000196628	TCF4	transcription factor 4 [Source:HGNC Symbol;Acc:11634]	5.78E-05	-0.280349
ENSG00000114854	TNNC1	troponin C type 1 (slow) [Source:HGNC Symbol;Acc:11943]	5.87E-05	0.280113
ENSG00000101276	SLC52A3	solute carrier family 52 (riboflavin transporter), member 3 [Source:HGNC Symbol;Acc:16187]	5.99E-05	0.279807
ENSG00000101335	MYL9	myosin, light chain 9, regulatory [Source:HGNC Symbol;Acc:15754]	6.20E-05	0.279238
ENSG00000211957	IGHV3-35	immunoglobulin heavy variable 3-35 (non-functional) [Source:HGNC Symbol;Acc:5598]	6.23E-05	0.279169
ENSG00000155657	TTN	titin [Source:HGNC Symbol;Acc:12403]	6.27E-05	0.279079
ENSG00000164744	SUN3	Sad1 and UNC84 domain containing 3 [Source:HGNC Symbol;Acc:22429]	6.47E-05	0.278584
ENSG00000226479	TMEM185B	transmembrane protein 185B [Source:HGNC Symbol;Acc:18896]	6.64E-05	0.278164
ENSG00000126012	KDM5C	lysine (K)-specific demethylase 5C [Source:HGNC Symbol;Acc:11114]	6.70E-05	0.278013
ENSG00000058404	CAMK2B	calcium/calmodulin-dependent protein kinase II beta [Source:HGNC Symbol;Acc:1461]	6.72E-05	0.277966
ENSG00000172292	CERS6	ceramide synthase 6 [Source:HGNC Symbol;Acc:23826]	6.76E-05	0.277888
ENSG00000163380	LMOD3	leiomodin 3 (fetal) [Source:HGNC Symbol;Acc:6649]	6.78E-05	0.27783
ENSG00000136828	RALGPS1	Ral GEF with PH domain and SH3 binding motif 1 [Source:HGNC Symbol;Acc:16851]	6.98E-05	0.277359
ENSG00000204852	TCTN1	tectonic family member 1 [Source:HGNC Symbol;Acc:26113]	7.02E-05	-0.277276
ENSG00000147475	ERLIN2	ER lipid raft associated 2 [Source:HGNC Symbol;Acc:1356]	7.18E-05	0.276921
ENSG00000143543	JTB	jumping translocation breakpoint [Source:HGNC Symbol;Acc:6201]	7.26E-05	-0.27674
ENSG00000182263	FIGN	fidgetin [Source:HGNC Symbol;Acc:13285]	7.32E-05	0.276612

ENSG00000253270	AC090541.1		7.34E-05	-0.276573
ENSG00000151729	SLC25A4	solute carrier family 25 (mitochondrial carrier; adenine nucleotide translocator), member 4 [Source:HGNC Symbol;Acc:1	7.40E-05	0.27644
ENSG00000223930	AC109779.1		7.53E-05	0.27616
ENSG00000087301	TXNDC16	thioredoxin domain containing 16 [Source:HGNC Symbol;Acc:19965]	7.94E-05	-0.275311
ENSG00000214097	SMCO1	single-pass membrane protein with coiled-coil domains 1 [Source:HGNC Symbol;Acc:27407]	7.99E-05	0.275207
ENSG00000164440	TXLNB	taxilin beta [Source:HGNC Symbol;Acc:21617]	8.30E-05	0.274604
ENSG00000205710	C17orf107	chromosome 17 open reading frame 107 [Source:HGNC Symbol;Acc:37238]	8.45E-05	0.274314
ENSG00000233581	AC069155.1		8.75E-05	-0.273749
ENSG00000121769	FABP3	fatty acid binding protein 3, muscle and heart (mammary-derived growth inhibitor) [Source:HGNC Symbol;Acc:3557]	8.77E-05	0.273698
ENSG00000257643	AC084824.2		8.82E-05	-0.273623
ENSG00000072062	PRKACA	protein kinase, cAMP-dependent, catalytic, alpha [Source:HGNC Symbol;Acc:9380]	8.82E-05	0.273609
ENSG00000100170	SLC5A1	solute carrier family 5 (sodium/glucose cotransporter), member 1 [Source:HGNC Symbol;Acc:11036]	8.83E-05	0.273604
ENSG00000186432	KPNA4	karyopherin alpha 4 (importin alpha 3) [Source:HGNC Symbol;Acc:6397]	8.97E-05	0.273346
ENSG00000115993	TRAK2	trafficking protein, kinesin binding 2 [Source:HGNC Symbol;Acc:13206]	9.35E-05	0.272664
ENSG00000230621	AC068138.1		9.46E-05	-0.272487
ENSG00000163833	FBXO40	F-box protein 40 [Source:HGNC Symbol;Acc:29816]	9.62E-05	0.272198
ENSG00000011275	RNF216	ring finger protein 216 [Source:HGNC Symbol;Acc:21698]	9.70E-05	0.272083
ENSG00000104936	DMPK	dystrophia myotonica-protein kinase [Source:HGNC Symbol;Acc:2933]	9.82E-05	0.27188
ENSG00000042317	SPATA7	spermatogenesis associated 7 [Source:HGNC Symbol;Acc:20423]	0.0001023	-0.271215
ENSG00000170145	SIK2	salt-inducible kinase 2 [Source:HGNC Symbol;Acc:21680]	0.0001056	-0.270696
ENSG00000149294	NCAM1	neural cell adhesion molecule 1 [Source:HGNC Symbol;Acc:7656]	0.0001058	0.270664
ENSG00000132938	MTUS2	microtubule associated tumor suppressor candidate 2 [Source:HGNC Symbol;Acc:20595]	0.0001065	0.270549
ENSG00000185033	SEMA4B	sema domain, immunoglobulin domain (Ig), transmembrane domain (TM) and short cytoplasmic domain, (semaphorin)	0.0001104	-0.269966
ENSG00000146066	HIGD2A	HIG1 hypoxia inducible domain family, member 2A [Source:HGNC Symbol;Acc:28311]	0.0001139	0.269442
ENSG00000112531	QKI	QKI, KH domain containing, RNA binding [Source:HGNC Symbol;Acc:21100]	0.0001141	0.26942
ENSG00000126882	FAM78A	family with sequence similarity 78, member A [Source:HGNC Symbol;Acc:25465]	0.0001157	0.26919
ENSG00000109458	GAB1	GRB2-associated binding protein 1 [Source:HGNC Symbol;Acc:4066]	0.0001163	0.26911
ENSG00000260285	AL133367.1		0.0001189	0.268744
ENSG00000107862	GBF1	golgi brefeldin A resistant guanine nucleotide exchange factor 1 [Source:HGNC Symbol;Acc:4181]	0.0001193	0.268692
ENSG00000118729	CASQ2	calsequestrin 2 (cardiac muscle) [Source:HGNC Symbol;Acc:1513]	0.0001215	0.268387
ENSG00000142235	LMTK3	lemur tyrosine kinase 3 [Source:HGNC Symbol;Acc:19295]	0.0001222	0.26829
ENSG00000107771	CCSER2	coiled-coil serine-rich protein 2 [Source:HGNC Symbol;Acc:29197]	0.0001259	0.2678
ENSG00000183814	LIN9	lin-9 homolog (C. elegans) [Source:HGNC Symbol;Acc:30830]	0.0001278	0.267549
ENSG00000256043	CTSO	cathepsin O [Source:HGNC Symbol;Acc:2542]	0.0001309	0.267147
ENSG00000105954	NPVF	neuropeptide VF precursor [Source:HGNC Symbol;Acc:13782]	0.0001317	0.267046
ENSG00000254828	AP005597.1		0.0001322	-0.26699

ENSG00000179284	DAND5	DAN domain family member 5, BMP antagonist [Source:HGNC Symbol;Acc:26780]	0.0001342	0.266741
ENSG00000197696	NMB	neuromedin B [Source:HGNC Symbol;Acc:7842]	0.0001361	-0.266517
ENSG00000253339	AC111149.2		0.0001363	-0.266482
ENSG00000174099	MSRB3	methionine sulfoxide reductase B3 [Source:HGNC Symbol;Acc:27375]	0.0001404	0.265985
ENSG00000126003	PLAGL2	pleiomorphic adenoma gene-like 2 [Source:HGNC Symbol;Acc:9047]	0.0001456	0.265377
ENSG00000167703	SLC43A2	solute carrier family 43 (amino acid system L transporter), member 2 [Source:HGNC Symbol;Acc:23087]	0.0001457	-0.265382
ENSG00000261146	AC007159.1		0.0001499	0.264896
ENSG00000205560	CPT1B	carnitine palmitoyltransferase 1B (muscle) [Source:HGNC Symbol;Acc:2329]	0.0001516	0.264709
ENSG00000161217	PCYT1A	phosphate cytidylyltransferase 1, choline, alpha [Source:HGNC Symbol;Acc:8754]	0.0001582	0.263991
ENSG00000160703	NLRX1	NLR family member X1 [Source:HGNC Symbol;Acc:29890]	0.0001601	0.263797
ENSG00000239961	LILRA4	leukocyte immunoglobulin-like receptor, subfamily A (with TM domain), member 4 [Source:HGNC Symbol;Acc:15503]	0.0001635	-0.263443
ENSG00000120254	MTHFD1L	methylenetetrahydrofolate dehydrogenase (NADP+ dependent) 1-like [Source:HGNC Symbol;Acc:21055]	0.0001662	-0.263167
ENSG00000105953	OGDH	oxoglutarate (alpha-ketoglutarate) dehydrogenase (lipoamide) [Source:HGNC Symbol;Acc:8124]	0.0001667	0.263113
ENSG00000140545	MFGE8	milk fat globule-EGF factor 8 protein [Source:HGNC Symbol;Acc:7036]	0.0001698	0.262802
ENSG00000001461	NIPAL3	NIPA-like domain containing 3 [Source:HGNC Symbol;Acc:25233]	0.0001739	0.262398
ENSG00000137288	UQCC2	ubiquinol-cytochrome c reductase complex assembly factor 2 [Source:HGNC Symbol;Acc:21237]	0.0001751	0.262288
ENSG00000099203	TMED1	transmembrane emp24 protein transport domain containing 1 [Source:HGNC Symbol;Acc:17291]	0.0001776	0.262041
ENSG00000267060	PTGES3L	prostaglandin E synthase 3 (cytosolic)-like [Source:HGNC Symbol;Acc:43943]	0.0001777	0.262039
ENSG00000205138	SDHAF1	succinate dehydrogenase complex assembly factor 1 [Source:HGNC Symbol;Acc:33867]	0.000179	-0.261913
ENSG00000101400	SNTA1	syntrophin, alpha 1 [Source:HGNC Symbol;Acc:11167]	0.0001797	0.261845
ENSG00000130382	MLLT1	myeloid/lymphoid or mixed-lineage leukemia (trithorax homolog, Drosophila); translocated to, 1 [Source:HGNC Symbol;	0.0001807	-0.261751
ENSG00000110237	ARHGEF17	Rho guanine nucleotide exchange factor (GEF) 17 [Source:HGNC Symbol;Acc:21726]	0.0001811	0.261712
ENSG00000184575	XPOT	exportin, tRNA [Source:HGNC Symbol;Acc:12826]	0.0001817	-0.261661
ENSG00000204271	SPIN3	spindlin family, member 3 [Source:HGNC Symbol;Acc:27272]	0.0001821	-0.261627
ENSG00000172780	RAB43	RAB43, member RAS oncogene family [Source:HGNC Symbol;Acc:19983]	0.0001837	-0.261479
ENSG00000184007	PTP4A2	protein tyrosine phosphatase type IVA, member 2 [Source:HGNC Symbol;Acc:9635]	0.0001872	-0.261148
ENSG00000020577	SAMD4A	sterile alpha motif domain containing 4A [Source:HGNC Symbol;Acc:23023]	0.0001943	0.260522
ENSG00000231816			0.0001976	-0.260239
ENSG00000131089	ARHGEF9	Cdc42 guanine nucleotide exchange factor (GEF) 9 [Source:HGNC Symbol;Acc:14561]	0.0001995	0.260069
ENSG00000100445	SDR39U1	short chain dehydrogenase/reductase family 39U, member 1 [Source:HGNC Symbol;Acc:20275]	0.0002051	0.259595
ENSG00000139832	RAB20	RAB20, member RAS oncogene family [Source:HGNC Symbol;Acc:18260]	0.0002063	-0.259502
ENSG00000146013	GFRA3	GDNF family receptor alpha 3 [Source:HGNC Symbol;Acc:4245]	0.0002075	0.259408
ENSG00000211455	STK38L	serine/threonine kinase 38 like [Source:HGNC Symbol;Acc:17848]	0.000208	0.25936
ENSG00000064393	HIPK2	homeodomain interacting protein kinase 2 [Source:HGNC Symbol;Acc:14402]	0.0002087	0.259304
ENSG00000230544			0.0002102	0.259185
ENSG00000119401	TRIM32	tripartite motif containing 32 [Source:HGNC Symbol;Acc:16380]	0.0002106	0.259146

ENSG00000182963	GJC1	gap junction protein, gamma 1, 45kDa [Source:HGNC Symbol;Acc:4280]	0.0002154	0.258761
ENSG00000186803	IFNA10	interferon, alpha 10 [Source:HGNC Symbol;Acc:5418]	0.0002161	-0.258701
ENSG00000201583	RN7SKP191	RNA, 7SK small nuclear pseudogene 191 [Source:HGNC Symbol;Acc:45915]	0.0002161	-0.258701
ENSG00000206741	Y_RNA	Y RNA [Source:RFAM;Acc:RF00019]	0.0002161	-0.258701
ENSG00000228365	AL133351.2		0.0002161	-0.258701
ENSG00000235572	AL353615.1		0.0002161	-0.258701
ENSG00000238511		Small nucleolar RNA U13 [Source:RFAM;Acc:RF01210]	0.0002161	-0.258701
ENSG00000248973	AC106799.2		0.0002161	-0.258701
ENSG00000257662	EIF4A1P12	eukaryotic translation initiation factor 4A1 pseudogene 12 [Source:HGNC Symbol;Acc:37933]	0.0002161	-0.258701
ENSG00000259136	AL137100.2		0.0002161	-0.258701
ENSG00000262107	MTND6P33		0.0002161	-0.258701
ENSG00000264127	SCML2P1	sex comb on midleg-like 2 (Drosophila) pseudogene 1 [Source:HGNC Symbol;Acc:44329]	0.0002161	-0.258701
ENSG00000264421	AC007448.2		0.0002161	-0.258701
ENSG00000228075	BOD1L2	biorientation of chromosomes in cell division 1-like 2 [Source:HGNC Symbol;Acc:28505]	0.0002196	-0.258428
ENSG00000212135	SNORD67	small nucleolar RNA, C/D box 67 [Source:HGNC Symbol;Acc:32728]	0.0002203	-0.258377
ENSG00000166278	C2	complement component 2 [Source:HGNC Symbol;Acc:1248]	0.0002245	0.258054
ENSG00000231645	KRT17P6		0.0002246	-0.258043
ENSG00000174206	C12orf66	chromosome 12 open reading frame 66 [Source:HGNC Symbol;Acc:26517]	0.0002274	0.257828
ENSG00000168497	CAVIN2	serum deprivation response [Source:HGNC Symbol;Acc:10690]	0.0002335	0.257378
ENSG00000226900	AL451069.1		0.000236	0.257199
ENSG00000158270	COLEC12	collectin sub-family member 12 [Source:HGNC Symbol;Acc:16016]	0.0002418	0.25678
ENSG00000179965	ZNF771	zinc finger protein 771 [Source:HGNC Symbol;Acc:29653]	0.0002424	-0.256733
ENSG00000213445	SIPA1	signal-induced proliferation-associated 1 [Source:HGNC Symbol;Acc:10885]	0.0002431	-0.256676
ENSG00000198336	MYL4	myosin, light chain 4, alkali; atrial, embryonic [Source:HGNC Symbol;Acc:7585]	0.0002433	0.256667
ENSG00000023330	ALAS1	aminolevulinate, delta-, synthase 1 [Source:HGNC Symbol;Acc:396]	0.0002436	0.256644
ENSG00000131462	TUBG1	tubulin, gamma 1 [Source:HGNC Symbol;Acc:12417]	0.0002487	0.256291
ENSG00000239696	AC092104.1		0.0002577	-0.255669
ENSG00000090266	NDUFB2	NADH dehydrogenase (ubiquinone) 1 beta subcomplex, 2, 8kDa [Source:HGNC Symbol;Acc:7697]	0.0002595	0.255551
ENSG00000186532	SMYD4	SET and MYND domain containing 4 [Source:HGNC Symbol;Acc:21067]	0.0002617	-0.255404
ENSG00000106025	TSPAN12	tetraspanin 12 [Source:HGNC Symbol;Acc:21641]	0.0002655	0.255148
ENSG00000135636	DYSF	dysferlin [Source:HGNC Symbol;Acc:3097]	0.0002665	0.255088
ENSG00000147642	SYBU	syntabulin (syntaxin-interacting) [Source:HGNC Symbol;Acc:26011]	0.0002752	0.254533
ENSG00000147548	NSD3	Wolf-Hirschhorn syndrome candidate 1-like 1 [Source:HGNC Symbol;Acc:12767]	0.0002754	-0.254512
ENSG00000184949	FAM227A	family with sequence similarity 227, member A [Source:HGNC Symbol;Acc:44197]	0.0002758	-0.254494
ENSG00000146729	NIPSNAP2	glioblastoma amplified sequence [Source:HGNC Symbol;Acc:4179]	0.0002777	0.254371
ENSG00000127533	F2RL3	coagulation factor II (thrombin) receptor-like 3 [Source:HGNC Symbol;Acc:3540]	0.0002789	-0.2543

ENSG00000134853	PDGFRA	platelet-derived growth factor receptor, alpha polypeptide [Source:HGNC Symbol;Acc:8803]	0.0002816	0.254132
ENSG00000136270	TBRG4	transforming growth factor beta regulator 4 [Source:HGNC Symbol;Acc:17443]	0.0002891	0.253667
ENSG00000213801	ZNF321P	ZNF816-ZNF321P readthrough [Source:HGNC Symbol;Acc:38879]	0.0002915	-0.253524
ENSG00000121797	CCRL2	chemokine (C-C motif) receptor-like 2 [Source:HGNC Symbol;Acc:1612]	0.0002916	-0.253523
ENSG00000260126	AC092326.1		0.0002928	-0.253445
ENSG00000099875	MKNK2	MAP kinase interacting serine/threonine kinase 2 [Source:HGNC Symbol;Acc:7111]	0.0002971	-0.25319
ENSG00000147601	TERF1	telomeric repeat binding factor (NIMA-interacting) 1 [Source:HGNC Symbol;Acc:11728]	0.0002975	-0.253163
ENSG00000136653		Ras association (RalGDS/AF-6) domain family member 5 [Source:HGNC Symbol;Acc:17609]	0.0003004	-0.252998
ENSG00000170807	LMOD2	leiomodin 2 (cardiac) [Source:HGNC Symbol;Acc:6648]	0.0003055	0.252704
ENSG00000088882	CPXM1	carboxypeptidase X (M14 family), member 1 [Source:HGNC Symbol;Acc:15771]	0.0003062	0.252667
ENSG00000080845	DLGAP4	discs, large (Drosophila) homolog-associated protein 4 [Source:HGNC Symbol;Acc:24476]	0.0003089	0.252507
ENSG00000004487	KDM1A	lysine (K)-specific demethylase 1A [Source:HGNC Symbol;Acc:29079]	0.0003128	-0.252287
ENSG00000119682	AREL1	apoptosis resistant E3 ubiquitin protein ligase 1 [Source:HGNC Symbol;Acc:20363]	0.0003159	0.252112
ENSG00000089486	CDIP1	cell death-inducing p53 target 1 [Source:HGNC Symbol;Acc:13234]	0.0003234	0.251696
ENSG00000129245	FXR2	fragile X mental retardation, autosomal homolog 2 [Source:HGNC Symbol;Acc:4024]	0.0003242	0.251654
ENSG00000114770	ABCC5	ATP-binding cassette, sub-family C (CFTR/MRP), member 5 [Source:HGNC Symbol;Acc:56]	0.0003258	-0.251565
ENSG00000170043	TRAPPC1	trafficking protein particle complex 1 [Source:HGNC Symbol;Acc:19894]	0.0003356	0.251045
ENSG00000232556	AC092570.2		0.0003388	-0.250877
ENSG00000171161	ZNF672	zinc finger protein 672 [Source:HGNC Symbol;Acc:26179]	0.000339	0.250867
ENSG00000150457	LATS2	large tumor suppressor kinase 2 [Source:HGNC Symbol;Acc:6515]	0.0003394	0.250847
ENSG00000022267	FHL1	four and a half LIM domains 1 [Source:HGNC Symbol;Acc:3702]	0.0003398	0.250828
ENSG00000169692	AGPAT2	1-acylglycerol-3-phosphate O-acyltransferase 2 [Source:HGNC Symbol;Acc:325]	0.0003419	-0.250718
ENSG00000148677	ANKRD1	ankyrin repeat domain 1 (cardiac muscle) [Source:HGNC Symbol;Acc:15819]	0.0003456	0.25053
ENSG00000178104	PDE4DIP	phosphodiesterase 4D interacting protein [Source:HGNC Symbol;Acc:15580]	0.0003457	0.250518
ENSG00000249145	LINC02517		0.0003587	0.249872
ENSG00000185515	BRCC3	BRCA1/BRCA2-containing complex, subunit 3 [Source:HGNC Symbol;Acc:24185]	0.0003607	-0.249766
ENSG00000141424	SLC39A6	solute carrier family 39 (zinc transporter), member 6 [Source:HGNC Symbol;Acc:18607]	0.0003614	-0.249734
ENSG00000078668	VDAC3	voltage-dependent anion channel 3 [Source:HGNC Symbol;Acc:12674]	0.0003616	0.249722
ENSG00000104047	DTWD1	DTW domain containing 1 [Source:HGNC Symbol;Acc:30926]	0.0003713	-0.249253
ENSG00000067225	PKM	pyruvate kinase, muscle [Source:HGNC Symbol;Acc:9021]	0.0003714	0.249244
ENSG00000241935	HOGA1	4-hydroxy-2-oxoglutarate aldolase 1 [Source:HGNC Symbol;Acc:25155]	0.0003795	0.248866
ENSG00000166484	MAPK7	mitogen-activated protein kinase 7 [Source:HGNC Symbol;Acc:6880]	0.0003842	0.248642
ENSG00000154305	MIA3	melanoma inhibitory activity family, member 3 [Source:HGNC Symbol;Acc:24008]	0.0003861	-0.248556
ENSG00000250392	LINC02502		0.0004006	0.247901
ENSG00000186994	KANK3	KN motif and ankyrin repeat domains 3 [Source:HGNC Symbol;Acc:24796]	0.0004007	-0.247892
ENSG00000163110	PDLIM5	PDZ and LIM domain 5 [Source:HGNC Symbol;Acc:17468]	0.0004027	0.247804

ENSG00000260176	AC141586.2		0.0004057	-0.24767
ENSG00000136783	NIPSNAP3A	nipsnap homolog 3A (C. elegans) [Source:HGNC Symbol;Acc:23619]	0.0004067	0.247629
ENSG00000006327	TNFRSF12A	tumor necrosis factor receptor superfamily, member 12A [Source:HGNC Symbol;Acc:18152]	0.0004177	0.247154
ENSG00000108861	DUSP3	dual specificity phosphatase 3 [Source:HGNC Symbol;Acc:3069]	0.0004197	0.24706
ENSG00000014641	MDH1	malate dehydrogenase 1, NAD (soluble) [Source:HGNC Symbol;Acc:6970]	0.0004201	0.247044
ENSG00000263082			0.0004312	-0.246576
ENSG00000053108	FSTL4	follistatin-like 4 [Source:HGNC Symbol;Acc:21389]	0.0004336	0.24648
ENSG00000071967	CYBRD1	cytochrome b reductase 1 [Source:HGNC Symbol;Acc:20797]	0.0004368	0.246343
ENSG00000104818	CGB2	chorionic gonadotropin, beta polypeptide 2 [Source:HGNC Symbol;Acc:16722]	0.0004416	-0.246143
ENSG00000078804	TP53INP2	tumor protein p53 inducible nuclear protein 2 [Source:HGNC Symbol;Acc:16104]	0.0004422	0.246121
ENSG00000070388	FGF22	fibroblast growth factor 22 [Source:HGNC Symbol;Acc:3679]	0.0004509	-0.245773
ENSG00000117010	ZNF684	zinc finger protein 684 [Source:HGNC Symbol;Acc:28418]	0.0004556	0.245584
ENSG00000255409	RSF1-IT1	RSF1 intronic transcript 1 (non-protein coding) [Source:HGNC Symbol;Acc:41439]	0.0004673	0.245128
ENSG00000003509	NDUFAF7	NADH dehydrogenase (ubiquinone) complex I, assembly factor 7 [Source:HGNC Symbol;Acc:28816]	0.0004729	-0.244906
ENSG00000143891	GALM	galactose mutarotase (aldose 1-epimerase) [Source:HGNC Symbol;Acc:24063]	0.0004787	-0.244688
ENSG00000244021	AC093591.1		0.0004804	-0.244627
ENSG00000116237	ICMT	isoprenylcysteine carboxyl methyltransferase [Source:HGNC Symbol;Acc:5350]	0.000482	0.244563
ENSG00000170027	YWHAG	tyrosine 3-monooxygenase/tryptophan 5-monooxygenase activation protein, gamma [Source:HGNC Symbol;Acc:12852]	0.0004879	0.24434
ENSG00000118946	PCDH17	protocadherin 17 [Source:HGNC Symbol;Acc:14267]	0.000493	-0.244157
ENSG00000117118	SDHB	succinate dehydrogenase complex, subunit B, iron sulfur (Ip) [Source:HGNC Symbol;Acc:10681]	0.0004971	0.244002
ENSG00000104472	CHRAC1	chromatin accessibility complex 1 [Source:HGNC Symbol;Acc:13544]	0.000499	0.243933
ENSG00000239739	AC026316.2		0.0004999	-0.243898
ENSG00000259401			0.0005007	-0.243876
ENSG00000168675	LDLRAD4	low density lipoprotein receptor class A domain containing 4 [Source:HGNC Symbol;Acc:1224]	0.0005021	-0.243825
ENSG00000148572	NRBF2	nuclear receptor binding factor 2 [Source:HGNC Symbol;Acc:19692]	0.0005119	-0.243466
ENSG00000184144	CNTN2	contactin 2 (axonal) [Source:HGNC Symbol;Acc:2172]	0.0005138	0.243406
ENSG00000080603	SRCAP	Snf2-related CREBBP activator protein [Source:HGNC Symbol;Acc:16974]	0.0005162	0.243315
ENSG00000172159	FRMD3	FERM domain containing 3 [Source:HGNC Symbol;Acc:24125]	0.0005175	0.243271
ENSG00000114923	SLC4A3	solute carrier family 4 (anion exchanger), member 3 [Source:HGNC Symbol;Acc:11029]	0.000523	0.243076
ENSG00000137547	MRPL15	mitochondrial ribosomal protein L15 [Source:HGNC Symbol;Acc:14054]	0.0005252	0.242999
ENSG00000175334	BANF1	barrier to autointegration factor 1 [Source:HGNC Symbol;Acc:17397]	0.0005308	0.242806
ENSG00000019144	PHLDB1	pleckstrin homology-like domain, family B, member 1 [Source:HGNC Symbol;Acc:23697]	0.0005418	0.242432
ENSG00000157680	DGKI	diacylglycerol kinase, iota [Source:HGNC Symbol;Acc:2855]	0.0005538	0.242034
ENSG00000196507	TCEAL3	transcription elongation factor A (SII)-like 3 [Source:HGNC Symbol;Acc:28247]	0.0005592	-0.241851
ENSG00000115221	ITGB6	integrin, beta 6 [Source:HGNC Symbol;Acc:6161]	0.0005603	0.24182
ENSG00000267472	AC005332.3		0.0005655	-0.241652

ENSG00000197296	FITM2	fat storage-inducing transmembrane protein 2 [Source:HGNC Symbol;Acc:16135]	0.0005701	0.241497
ENSG00000116017	ARID3A	AT rich interactive domain 3A (BRIGHT-like) [Source:HGNC Symbol;Acc:3031]	0.0005728	-0.241413
ENSG00000267141	AC012615.4		0.000573	0.24141
ENSG00000250635	CXXC5-AS1		0.0005811	0.241151
ENSG00000100122	CRYBB1	crystallin, beta B1 [Source:HGNC Symbol;Acc:2397]	0.0005884	0.24092
ENSG00000126858	RHOT1	ras homolog family member T1 [Source:HGNC Symbol;Acc:21168]	0.0005927	-0.24078
ENSG00000205678	TECRL	trans-2,3-enoyl-CoA reductase-like [Source:HGNC Symbol;Acc:27365]	0.0005956	0.240692
ENSG00000198759	EGFL6	EGF-like-domain, multiple 6 [Source:HGNC Symbol;Acc:3235]	0.0006181	0.240011
ENSG00000145349	CAMK2D	calcium/calmodulin-dependent protein kinase II delta [Source:HGNC Symbol;Acc:1462]	0.0006266	0.239754
ENSG00000244544	RN7SL446P	RNA, 7SL, cytoplasmic 446, pseudogene [Source:HGNC Symbol;Acc:46462]	0.0006335	-0.239551
ENSG00000197794	IGKV7-3	immunoglobulin kappa variable 7-3 (pseudogene) [Source:HGNC Symbol;Acc:5839]	0.0006338	-0.239542
ENSG00000231604	AL133232.1		0.0006341	-0.239533
ENSG00000073910	FRY	furry homolog (Drosophila) [Source:HGNC Symbol;Acc:20367]	0.0006389	0.239393
ENSG00000077254	USP33	ubiquitin specific peptidase 33 [Source:HGNC Symbol;Acc:20059]	0.0006442	-0.239242
ENSG00000196588	MKL1	megakaryoblastic leukemia (translocation) 1 [Source:HGNC Symbol;Acc:14334]	0.0006513	0.239036
ENSG00000140682	TGFB1I1	transforming growth factor beta 1 induced transcript 1 [Source:HGNC Symbol;Acc:11767]	0.0006583	0.238844
ENSG00000232230	TPM4P1	tropomyosin 4 pseudogene 1 [Source:HGNC Symbol;Acc:45234]	0.0006696	0.238521
ENSG00000180785	OR51E1	olfactory receptor, family 51, subfamily E, member 1 [Source:HGNC Symbol;Acc:15194]	0.00067	0.238518
ENSG00000264916	RN7SL230P	RNA, 7SL, cytoplasmic 230, pseudogene [Source:HGNC Symbol;Acc:46246]	0.000671	-0.238483
ENSG00000119333	WDR34	WD repeat domain 34 [Source:HGNC Symbol;Acc:28296]	0.0006766	0.23833
ENSG00000261666	LINC00560	long intergenic non-protein coding RNA 560 [Source:HGNC Symbol;Acc:43704]	0.000686	-0.238078
ENSG00000180628	PCGF5	polycomb group ring finger 5 [Source:HGNC Symbol;Acc:28264]	0.0006928	0.237888
ENSG00000128309	MPST	mercaptopyruvate sulfurtransferase [Source:HGNC Symbol;Acc:7223]	0.0007023	-0.237633
ENSG00000186191	BPIFB4	BPI fold containing family B, member 4 [Source:HGNC Symbol;Acc:16179]	0.0007033	0.237612
ENSG00000225485		Rho GTPase activating protein 23 [Source:HGNC Symbol;Acc:29293]	0.0007314	0.236874
ENSG00000204564	C6orf136	chromosome 6 open reading frame 136 [Source:HGNC Symbol;Acc:21301]	0.0007334	0.236824
ENSG00000070610	GBA2	glucosidase, beta (bile acid) 2 [Source:HGNC Symbol;Acc:18986]	0.0007352	0.236778
ENSG00000177469	CAVIN1	polymerase I and transcript release factor [Source:HGNC Symbol;Acc:9688]	0.0007418	0.23661
ENSG00000269964	MEI4	meiosis-specific 4 homolog (S. cerevisiae) [Source:HGNC Symbol;Acc:43638]	0.0007485	0.236445
ENSG00000170430	MGMT	O-6-methylguanine-DNA methyltransferase [Source:HGNC Symbol;Acc:7059]	0.0007597	0.236168
ENSG00000124701	APOBEC2	apolipoprotein B mRNA editing enzyme, catalytic polypeptide-like 2 [Source:HGNC Symbol;Acc:605]	0.0007627	0.236091
ENSG00000185621	LMLN	leishmanolysin-like (metallopeptidase M8 family) [Source:HGNC Symbol;Acc:15991]	0.0007672	-0.235977
ENSG00000119820	YIPF4	Yip1 domain family, member 4 [Source:HGNC Symbol;Acc:28145]	0.0007703	0.235902
ENSG00000146926	ASB10	ankyrin repeat and SOCS box containing 10 [Source:HGNC Symbol;Acc:17185]	0.000771	0.235889
ENSG00000107872	FBXL15	F-box and leucine-rich repeat protein 15 [Source:HGNC Symbol;Acc:28155]	0.0007821	-0.235618
ENSG00000188229	TUBB4B	tubulin, beta 4B class IVb [Source:HGNC Symbol;Acc:20771]	0.0008125	0.234901

ENSG00000113328	CCNG1	cyclin G1 [Source:HGNC Symbol;Acc:1592]	0.0008167	-0.2348
ENSG00000235194	PPP1R3E	protein phosphatase 1, regulatory subunit 3E [Source:HGNC Symbol;Acc:14943]	0.0008239	-0.234633
ENSG00000260124			0.0008242	0.234632
ENSG00000100412	ACO2	aconitase 2, mitochondrial [Source:HGNC Symbol;Acc:118]	0.0008248	0.234613
ENSG00000260624	AC018943.1		0.0008258	-0.234591
ENSG00000256269	HMBS	hydroxymethylbilane synthase [Source:HGNC Symbol;Acc:4982]	0.0008321	0.234447
ENSG00000188505	NCCRP1	non-specific cytotoxic cell receptor protein 1 homolog (zebrafish) [Source:HGNC Symbol;Acc:33739]	0.0008326	-0.23444
ENSG00000152661	GJA1	gap junction protein, alpha 1, 43kDa [Source:HGNC Symbol;Acc:4274]	0.0008415	0.234238
ENSG00000141564	RPTOR	regulatory associated protein of MTOR, complex 1 [Source:HGNC Symbol;Acc:30287]	0.0008447	0.234161
ENSG00000141026	MED9	mediator complex subunit 9 [Source:HGNC Symbol;Acc:25487]	0.0008481	0.234085
ENSG00000147124	ZNF41	zinc finger protein 41 [Source:HGNC Symbol;Acc:13107]	0.0008491	0.234062
ENSG00000159593	NAE1	NEDD8 activating enzyme E1 subunit 1 [Source:HGNC Symbol;Acc:621]	0.0008511	-0.234018
ENSG00000215450	AL022342.1		0.0008566	0.233902
ENSG00000223501	VPS52	vacuolar protein sorting 52 homolog (S. cerevisiae) [Source:HGNC Symbol;Acc:10518]	0.000858	0.233864
ENSG00000115593	SMYD1	SET and MYND domain containing 1 [Source:HGNC Symbol;Acc:20986]	0.0008623	0.233773
ENSG00000105676	ARMC6	armadillo repeat containing 6 [Source:HGNC Symbol;Acc:25049]	0.0008624	0.233768
ENSG00000109390	NDUFC1	NADH dehydrogenase (ubiquinone) 1, subcomplex unknown, 1, 6kDa [Source:HGNC Symbol;Acc:7705]	0.0008657	0.233695
ENSG00000157540	DYRK1A	dual-specificity tyrosine-(Y)-phosphorylation regulated kinase 1A [Source:HGNC Symbol;Acc:3091]	0.0008671	0.233664
ENSG00000154415	PPP1R3A	protein phosphatase 1, regulatory subunit 3A [Source:HGNC Symbol;Acc:9291]	0.000873	0.233541
ENSG00000260047	BCAP31P2		0.0008781	0.233429
ENSG00000149636	DSN1	DSN1, MIS12 kinetochore complex component [Source:HGNC Symbol;Acc:16165]	0.0008822	-0.233338
ENSG00000254370	AC025871.2		0.0008859	-0.233263
ENSG00000103175	WFDC1	WAP four-disulfide core domain 1 [Source:HGNC Symbol;Acc:15466]	0.0008894	-0.233186
ENSG00000154640	BTG3	BTG family, member 3 [Source:HGNC Symbol;Acc:1132]	0.0008996	-0.232968
ENSG00000225270	CICP12	capicua transcriptional repressor pseudogene 12 [Source:HGNC Symbol;Acc:37905]	0.0009061	-0.232828
ENSG00000160539	PLPP7	phosphatidic acid phosphatase type 2 domain containing 3 [Source:HGNC Symbol;Acc:28174]	0.0009083	0.232782
ENSG00000205403	CFI	complement factor I [Source:HGNC Symbol;Acc:5394]	0.0009104	0.232743
ENSG00000204178	TMEM57	transmembrane protein 57 [Source:HGNC Symbol;Acc:25572]	0.0009105	-0.232735
ENSG00000203356	LINC01562		0.0009128	0.232693
ENSG00000227560	RPS15AP30		0.0009188	0.232568
ENSG00000264514	AP000915.1		0.0009203	0.232536
ENSG00000109794	FAM149A	family with sequence similarity 149, member A [Source:HGNC Symbol;Acc:24527]	0.000948	-0.231969
ENSG00000250988	SNHG21		0.0009574	-0.231781
ENSG00000244146	AC106872.3		0.0009637	-0.231651
ENSG00000105429	MEGF8	multiple EGF-like-domains 8 [Source:HGNC Symbol;Acc:3233]	0.0009733	0.23146
ENSG00000126953	TIMM8A	translocase of inner mitochondrial membrane 8 homolog A (yeast) [Source:HGNC Symbol;Acc:11817]	0.0009764	0.231401

ENSG00000249035	CLMAT3		0.0009817	0.2313
ENSG00000174456	C12orf76	chromosome 12 open reading frame 76 [Source:HGNC Symbol;Acc:33790]	0.0009884	-0.231165
ENSG00000184113	CLDN5	claudin 5 [Source:HGNC Symbol;Acc:2047]	0.0009955	-0.231032
