# Supplementary material for: Transcriptomic Insights into the Atrial Fibrillation Susceptibility Locus near the MYOZ1 and SYNPO2L Genes
Source: Int J Mol Sci. 2024 Sep 25;25(19):10309. doi: 10.3390/ijms251910309 (PMC11477451; doi:10.3390/ijms251910309)
Supplement: Supplementary file 1 [file ijms-25-10309-s001.zip › SYNPO2L novel transcript first splice donor.pdf]

SYNPO2L transcript novel first splice donor

CCTGGCTGGCAGTGGCTGCCTGAGCT
CTCTACGTAGAGACAGTTCTGACCCCTGAAGAAGCCCACCCCACTACCATCCCCAGCTTGGGGTAAATAA
TATATTTAGCTGGCGACCTTGAGGTATCCTAAAGATTTGGAGTGTTGCCTTGGAGAACAACACTCTAACT
TTTCCTGCATCTTGGATGGAGACTACCCCTGACT
GTGAGTAGCAGGGGTGTGAAGCCCAGAGGACTGCCCTGGCAGTTGGA
AGGCCGAGATCAACTTTAACTTCTGTCTAAACTGTTAGTAACAGGGTCTCTGCAGGGAGG
TCTCTGGGAAGAAGGGCCACCTAGAGCATGTAGGTGTGTGAATGTATTTGAGTGATGGTG
TTTTGATGTGCACACATGTGTATTTACATAAGTGTATGTGAATGGAGCTGTATGTAAGCT
GGGGTATATGTGAATGAGAGAGAAAAGTAAATAGGAGAAAGTACGCCTACAGTTGGCTTT
CACTAGATGGATATTCTTGTTGAAGTGCTGTGTGGTGGGAGTTATTTCTGTGAATGTGTA
TGATGTGGATTGCATGAGGAGGGTGGAATTGTGAATGAAGATCCTCATTGCTAAGGGTTC
TTTGTGCTCATATCATAGGGGTAAGGGGTAGTGGAGAGGATGAGTCCTAAGCTTCATGAG
AAGCCTCCTAACATGGTCCCTATGATGGGTGGAAAGTGTTCCTTCTCATCTTTCCTCTCC
CTGGGACCTGCTTATCTCCCTGTCTGCCACGGAGAGTGACACATCCTTCTCCTCCCCCTC
CCATTGCCAGTCAGGAATGGGAGGCAGGTGAGGAATGTGTGTGGGGTGGATAAGAGCACA
TCTCTTGGAAGATAGGAGCAGAATAGCAGTAAAATGGGATGGAAGAGCTGTGGTCAGAGC
AGTGTCAGGGCAGCCAACTGGACTCTGCCTTATCTTTAACTGTCCTTGGTTTCACCTTTC
ACCCTCCACTCTGCCCCTAAGTCAGAGGACTTTGTCACAGGGGACATTGTGGGTCGGGTC
AGTGCAAGAGGGAGCTTGCTGGGATGGGGGGTGAAAGCCTTGGGGAACAGGACTAAAAGA
GGCTGGGAAGCCCCCCTGACCATTTTACCTCTTGGCCTCCCCAG

AACTGCAACGTGCAGAGAGCCTCCAAGAGAAGAGCA
TAAAAGAGGCCAAGACCAAATGCAGGACAATTGCATCCCTGCTCACTGCAGCCCCCAACCCCCACTCCAA
AGGGGTACTTATGTTTAAGAAACGGCGGCAGAGAGCCAAGAAGTACACCCTGGTGAGCTTCGGGGCTGCT
GCTGGGACAGGCGCTGAGGAGGAGGACGGCGTTCCCCCCACGAGTGAGTCCGAGCTGGACGAAGAAGCCT
TCTCTGACGCCCGCAGCCTCACCAATCAATCTGACTGGGACAGTCCCTATCTGGACATGGAGCTTGCCAG
GGCGGGCTCAAGAGCATCAGAGGGCCAGGGCTCTGGGCTGGGAGGGCAGCTGAGTGAGGTCTCTGGGCGA
GGGGTGCAGCTCTTTGAACAGCAGCGCCAGCGCGCAGACTCCAGCACCCAGGAACTGGCACGGGTCGAAC
CAGCAGCCATGCTCAACGGGGAAGGCCTGCAGTCACCACCTCGGGCCCAGAGTGCTCCCCCAGAGGCAGC
TGTGCTCCCACCCAGCCCCTTGCCGGCGCCTGTAGCCAGCCCCAGACCCTTCCAACCAGGTGGTGGAGCC
CCGACCCCAGCTCCAAGCATCTTTAACCGGTCAGCCAGGCCCTTTACCCCGGGCCTACAAGGGCAGCGGC
CAACTACCACCTCGGTTATTTTCCGGCCTTTAGCCCCCAAAAGGGCGAACGACAGCCTGGGGGGCCTCAG
CCCCGCCCCACCCCCCTTCTTGTCTTCGCAGGGGCCCACCCCTCTGCCCAGCTTCACTTCAGGGGTTCCC
AGCCACGCGCCAGTCTCTGGTTCCCCCAGCACCCCACGCTCCTCGGGCCCTGTGACAGCCACCAGCTCCC
TGTACATCCCAGCCCCTAGTCGGCCTGTCACCCCAGGTGGAGCTCCAGAGCCCCCCGCTCCTCCTAGCGC
AGCTGCCATGACCTCCACCGCTTCTATCTTCCTATCTGCGCCTTTGCGACCCTCTGCGCGCCCAGAGGCG
CCTGCCCCAGGCCCAGGGGCTCCTGAGCCCCCCAGCGCTCGCGAGCAGCGCATCTCTGTGCCAGCTGCCC
GCACGGGTATCCTGCAGGAGGCCCGGCGCCGGGGGACCCGGAAGCAGATGTTCCGGCCGGGAAAGGAGGA
GACGAAGAACTCGCCCAACCCCGAGCTGCTATCGCTGGTACAGAACCTGGATGAAAAGCCTCGGGCCGGG
GGTGCAGAATCTGGTCCTGAAGAAGATGCTCTGAGCCTCGGGGCTGAAGCCTGCAACTTCATGCAGCCAG
TAGGGGCCAGGAGTTACAAGACCCTGCCTCACGTGACACCTAAGACCCCCCCTCCAATGGCTCCCAAGAC
CCCGCCCCCTATGACTCCTAAGACTCCACCCCCAGTGGCTCCTAAGCCCCCATCTCGAGGGCTCCTTGAT
GGGCTCGTGAATGGGGCAGCCTCTTCGGCTGGAATCCCTGAGCCACCAAGGCTGCAGGGCAGGGGTGGGG
AGCTGTTTGCTAAGCGGCAGAGCCGTGCGGACAGGTATGTGGTGGAAGGTACACCTGGTCCTGGTCTTGG
CCCTCGGCCTAGAAGTCCTTCTCCTACCCCGTCTCTGCCCCCTTCCTGGAAATATTCACCCAACATCCGT
GCCCCGCCTCCTATTGCTTACAACCCACTGCTCTCTCCCTTTTTCCCCCAGGCGGCCCGAACTCTCCCTA
AGGCCCAATCCCAGGGGCCTCGGGCAACACCCAAGCAGGGCATCAAGGCTCTAGATTTTATGCGGCATCA
GCCCTATCAACTTAAAACTGCCATGTTCTGTTTTGATGAGGTTCCCCCGACTCCTGGCCCTATCGCCTCA
GGGTCCCCCAAAACTGCCCGAGTCCAGGAGATTCGCCGGTTTTCCACTCCGGCACCCCAGCCCACTGCAG
AACCCCTGGCTCCCACTGTGCTTGCCCCCCGAGCAGCCACTACACTGGATGAGCCCATCTGGAGAACAGA
ACTGGCCTCAGCCCCTGTTCCTAGCCCAGCCCCTCCTCCAGAGGCTCCCAGGGGCCTTGGGGCTTCTCCC
AGCTCCTGCGGTTTCCAGGTAGCCAGGCCCCGATTTTCAGCCACCAGAACAGGATTGCAAGCTCATGTGT
GGAGGCCTGGGGCAGGGCACCAGTGAACAGGCACAGGTCCCAGGACCAAGGAGAGGTGGAACATCCAGTT
CCTAAAGTTGCTTCTCCTACCCTATCCCATCCCCTGTCACGCATCTGGAAGCTAAATTGCCTCCTGCCAG
AGATGGTTTCCAAGTTGATGTCCCCTTCCCCCACCTTCCTCCTCACTCTCTACCTCCCTGCCGCTTTCCA

Commented [JS1]: Start of novel exon

Commented [JS2]: End of novel exon first splice donor

Commented [SJ3]: Beginning intron from novel exon first splice donor

Commented [SJ4]: Intron

Commented [JS5]: Start of common final exon

ACCAAGTATGTCTGCTTTGGTATCTTTGCCTCTCTTTGTCTCTGCATTTCCTTTCCTGGATCTCTGTCTT
TATTTCCAGGCTTCTCCACCCATATTCCTCCACAGATCTCTCTTCCTTGACATTTGTGCTTTTCTCCCTG
GGCCTCATTTTAATGTTCAGTGAGAAGTAAACAGAGCAGAAGTGACCACTGGGACTTCAGGCAAGAAGCT
CACCACCAGGCACACAGCAAAGGGACTGAACTGACCCCTGTTTGCACTAAGCCACCCCCACCCCCCACTC
TGCTTTCCCAAGCTTGACTGGCATATACCTAGGCCTGTGTGTGTGTGTGTGTGTGTGTGTGTGTGTGTGT
GTGTGCTCTTCCGTCTAAGGCATGAATAAGAGGGGAGGTCAAAATAAAGACCCAATCTGAGGCCGGGCAC
GGTGGCTCACGCCGGTAATCCCAGCACTTTGGGAGGCCGAGGCGGGCGGATCACGAGGTCAGGAGATCGA
GACCATCCTGGCTAACACGGTGAAACCCCATTTCCACTAAAAATACAAAAAATTAGCTGGGCGTGGTGGC
GAGCGCCTGTAGTCCCAGCTACTCGGGAGGCTGAGGCAGGAGAATGGCATGAACCTGGAAGGCGGAGCTT
GCAGTGAGCTGAGATTGCGCCACTGCACTCCAGCCTGGGCGACGGAGCGAGACTCTGTCTCAAAACAAAC
AAACAAACAAAAGACCCAATCTGAGTCTTATCGTTGTACTGATAGAAGGGTCAGATATCCCCACATGGAG
TTGAGTGGGAGAAAGAGATTCACTAGAGAATAACTCCTTAGAGACCAATGTCTGTAGCAGGTGTACAGCA
TCTTGTGAAAGTTATGGAGCATGAAAAGACTGAAGGGCCAGGACAGTTTGCATGGGCTGAGTTATACCAG
CTAGACCAGGAATAGAACAAAGAATTCTATACCTCAGGATTTCAAAAAGTTAGCAACTTGAGAGGCCAGT
GCTGAGCAACCCAGTACCCAGGAAATGAAAAAAAGAAAGAAAATTCCCTCCGAGAATGAACAAATCATTG
GCTTCATTGCCTCATGAGCTTGAGAGAAAGGAGAAGAGAGCCAGAGTGTGGCAAGTGAGGCCAAAATCAG
AAGCATGGCAGAAATGAGTGTAAGTGATTGAGCCACAGACAGAAGTGTGGCGAGGGACAATGCCATATTG
GGAGAAGGTAAAGTTGAGTAACAAGAAACCAACCGTGTGTGAGAGGGGGATTGGAAAAAAATTTGAGGGA
GAAGAATGTTAGAATGGAAGGGAATGATGGTGGAAGGGAGGTGTGAGGGTGTGTGCTGAGTGTTGAAAGA
ACGGTTGGTGTCTGTGTGATTTTCCTTGAGTCTGTTCTTCAGTGTGTCTTCTGCAGCTTGCCATGACTGC
CTGGGAAAGAGTAGGGAAATACCCAGAGCCAAAACCTCCTTTCAGTCCCACCCCATCCCTCAAAACCCCA
GCTATTGCTTCTTTTCAGCTTCAGGTCCTGATCTCCAATCTTAGTATGGACTCCCTTCTCACCAAGACCA
CCACCAGCTACGTTTGCTGTGTAATCTGGAAAGTGATAATTTCCTTTGCTTGTTGGGTGTGAGTCACAAT
ACTTTGGTTTGTGCACAAGAATAAATTTATGCCCCATACCTTC

SYNPO2L.N1 cDNA

First exon highlighted in yellow, second exon highlighted in blue, ATG start codon highlighted in green.

CCTGGCTGGCAGTGGCTGCCTGAGCTCTCTACGTAGAGACAGTTCTGACCCCTGAAGAAGCCCACCCCAC
TACCATCCCCAGCTTGGGGTAAATAATATATTTAGCTGGCGACCTTGAGGTATCCTAAAGATTTGGAGTG
TTGCCTTGGAGAACAACACTCTAACTTTTCCTGCATCTTGGATGGAGACTACCCCTGACTAACTGCAACG
TGCAGAGAGCCTCCAAGAGAAGAGCATAAAAGAGGCCAAGACCAAATGCAGGACAATTGCATCCCTGCTC
ACTGCAGCCCCCAACCCCCACTCCAAAGGGGTACTTATGTTTAAGAAACGGCGGCAGAGAGCCAAGAAGT
ACACCCTGGTGAGCTTCGGGGCTGCTGCTGGGACAGGCGCTGAGGAGGAGGACGGCGTTCCCCCCACGAG
TGAGTCCGAGCTGGACGAAGAAGCCTTCTCTGACGCCCGCAGCCTCACCAATCAATCTGACTGGGACAGT
CCCTATCTGGACATGGAGCTTGCCAGGGCGGGCTCAAGAGCATCAGAGGGCCAGGGCTCTGGGCTGGGAG
GGCAGCTGAGTGAGGTCTCTGGGCGAGGGGTGCAGCTCTTTGAACAGCAGCGCCAGCGCGCAGACTCCAG
CACCCAGGAACTGGCACGGGTCGAACCAGCAGCCATGCTCAACGGGGAAGGCCTGCAGTCACCACCTCGG
GCCCAGAGTGCTCCCCCAGAGGCAGCTGTGCTCCCACCCAGCCCCTTGCCGGCGCCTGTAGCCAGCCCCA
GACCCTTCCAACCAGGTGGTGGAGCCCCGACCCCAGCTCCAAGCATCTTTAACCGGTCAGCCAGGCCCTT
TACCCCGGGCCTACAAGGGCAGCGGCCAACTACCACCTCGGTTATTTTCCGGCCTTTAGCCCCCAAAAGG
GCGAACGACAGCCTGGGGGGCCTCAGCCCCGCCCCACCCCCCTTCTTGTCTTCGCAGGGGCCCACCCCTC
TGCCCAGCTTCACTTCAGGGGTTCCCAGCCACGCGCCAGTCTCTGGTTCCCCCAGCACCCCACGCTCCTC
GGGCCCTGTGACAGCCACCAGCTCCCTGTACATCCCAGCCCCTAGTCGGCCTGTCACCCCAGGTGGAGCT
CCAGAGCCCCCCGCTCCTCCTAGCGCAGCTGCCATGACCTCCACCGCTTCTATCTTCCTATCTGCGCCTT
TGCGACCCTCTGCGCGCCCAGAGGCGCCTGCCCCAGGCCCAGGGGCTCCTGAGCCCCCCAGCGCTCGCGA
GCAGCGCATCTCTGTGCCAGCTGCCCGCACGGGTATCCTGCAGGAGGCCCGGCGCCGGGGGACCCGGAAG
CAGATGTTCCGGCCGGGAAAGGAGGAGACGAAGAACTCGCCCAACCCCGAGCTGCTATCGCTGGTACAGA
ACCTGGATGAAAAGCCTCGGGCCGGGGGTGCAGAATCTGGTCCTGAAGAAGATGCTCTGAGCCTCGGGGC
TGAAGCCTGCAACTTCATGCAGCCAGTAGGGGCCAGGAGTTACAAGACCCTGCCTCACGTGACACCTAAG

ACCCCCCCTCCAATGGCTCCCAAGACCCCGCCCCCTATGACTCCTAAGACTCCACCCCCAGTGGCTCCTA
AGCCCCCATCTCGAGGGCTCCTTGATGGGCTCGTGAATGGGGCAGCCTCTTCGGCTGGAATCCCTGAGCC
ACCAAGGCTGCAGGGCAGGGGTGGGGAGCTGTTTGCTAAGCGGCAGAGCCGTGCGGACAGGTATGTGGTG
GAAGGTACACCTGGTCCTGGTCTTGGCCCTCGGCCTAGAAGTCCTTCTCCTACCCCGTCTCTGCCCCCTT
CCTGGAAATATTCACCCAACATCCGTGCCCCGCCTCCTATTGCTTACAACCCACTGCTCTCTCCCTTTTT
CCCCCAGGCGGCCCGAACTCTCCCTAAGGCCCAATCCCAGGGGCCTCGGGCAACACCCAAGCAGGGCATC
AAGGCTCTAGATTTTATGCGGCATCAGCCCTATCAACTTAAAACTGCCATGTTCTGTTTTGATGAGGTTC
CCCCGACTCCTGGCCCTATCGCCTCAGGGTCCCCCAAAACTGCCCGAGTCCAGGAGATTCGCCGGTTTTC
CACTCCGGCACCCCAGCCCACTGCAGAACCCCTGGCTCCCACTGTGCTTGCCCCCCGAGCAGCCACTACA
CTGGATGAGCCCATCTGGAGAACAGAACTGGCCTCAGCCCCTGTTCCTAGCCCAGCCCCTCCTCCAGAGG
CTCCCAGGGGCCTTGGGGCTTCTCCCAGCTCCTGCGGTTTCCAGGTAGCCAGGCCCCGATTTTCAGCCAC
CAGAACAGGATTGCAAGCTCATGTGTGGAGGCCTGGGGCAGGGCACCAGTGAACAGGCACAGGTCCCAGG
ACCAAGGAGAGGTGGAACATCCAGTTCCTAAAGTTGCTTCTCCTACCCTATCCCATCCCCTGTCACGCAT
CTGGAAGCTAAATTGCCTCCTGCCAGAGATGGTTTCCAAGTTGATGTCCCCTTCCCCCACCTTCCTCCTC
ACTCTCTACCTCCCTGCCGCTTTCCAACCAAGTATGTCTGCTTTGGTATCTTTGCCTCTCTTTGTCTCTG
CATTTCCTTTCCTGGATCTCTGTCTTTATTTCCAGGCTTCTCCACCCATATTCCTCCACAGATCTCTCTT
CCTTGACATTTGTGCTTTTCTCCCTGGGCCTCATTTTAATGTTCAGTGAGAAGTAAACAGAGCAGAAGTG
ACCACTGGGACTTCAGGCAAGAAGCTCACCACCAGGCACACAGCAAAGGGACTGAACTGACCCCTGTTTG
CACTAAGCCACCCCCACCCCCCACTCTGCTTTCCCAAGCTTGACTGGCATATACCTAGGCCTGTGTGTGT
GTGTGTGTGTGTGTGTGTGTGTGTGTGTGCTCTTCCGTCTAAGGCATGAATAAGAGGGGAGGTCAAAA
TAAAGACCCAATCTGAGGCCGGGCACGGTGGCTCACGCCGGTAATCCCAGCACTTTGGGAGGCCGAGGCG
GGCGGATCACGAGGTCAGGAGATCGAGACCATCCTGGCTAACACGGTGAAACCCCATTTCCACTAAAAAT
ACAAAAAATTAGCTGGGCGTGGTGGCGAGCGCCTGTAGTCCCAGCTACTCGGGAGGCTGAGGCAGGAGAA
TGGCATGAACCTGGAAGGCGGAGCTTGCAGTGAGCTGAGATTGCGCCACTGCACTCCAGCCTGGGCGACG
GAGCGAGACTCTGTCTCAAAACAAACAAACAAACAAAAGACCCAATCTGAGTCTTATCGTTGTACTGATA
GAAGGGTCAGATATCCCCACATGGAGTTGAGTGGGAGAAAGAGATTCACTAGAGAATAACTCCTTAGAGA
CCAATGTCTGTAGCAGGTGTACAGCATCTTGTGAAAGTTATGGAGCATGAAAAGACTGAAGGGCCAGGAC
AGTTTGCATGGGCTGAGTTATACCAGCTAGACCAGGAATAGAACAAAGAATTCTATACCTCAGGATTTCA
AAAAGTTAGCAACTTGAGAGGCCAGTGCTGAGCAACCCAGTACCCAGGAAATGAAAAAAAGAAAGAAAAT
TCCCTCCGAGAATGAACAAATCATTGGCTTCATTGCCTCATGAGCTTGAGAGAAAGGAGAAGAGAGCCAG
AGTGTGGCAAGTGAGGCCAAAATCAGAAGCATGGCAGAAATGAGTGTAAGTGATTGAGCCACAGACAGAA
GTGTGGCGAGGGACAATGCCATATTGGGAGAAGGTAAAGTTGAGTAACAAGAAACCAACCGTGTGTGAGA
GGGGGATTGGAAAAAAAATTTGAGGGAGAAGAATGTTAGAATGGAAGGGAATGATGGTGGAAGGGAGGTGT
GAGGGTGTGTGCTGAGTGTTGAAAGAACGGTTGGTGTCTGTGTGATTTTCCTTGAGTCTGTTCTTCAGTG
TGTCTTCTGCAGCTTGCCATGACTGCCTGGGAAAGAGTAGGGAAATACCCAGAGCCAAAACCTCCTTTCA
GTCCCACCCCATCCCTCAAAACCCCAGCTATTGCTTCTTTTCAGCTTCAGGTCCTGATCTCCAATCTTAG
TATGGACTCCCTTCTCACCAAGACCACCACCAGCTACGTTTGCTGTGTAATCTGGAAAGTGATAATTTCC
TTTGCTTGTTGGGTGTGAGTCACAATACTTTGGTTTGTGCACAAGAATAAATTTATGCCCCATACCTTC

Inferred AA sequence from first ATG

METTPD
