# Supplementary material for: Transcriptomic Insights into the Atrial Fibrillation Susceptibility Locus near the MYOZ1 and SYNPO2L Genes
Source: Int J Mol Sci. 2024 Sep 25;25(19):10309. doi: 10.3390/ijms251910309 (PMC11477451; doi:10.3390/ijms251910309)
Supplement: Supplementary file 1 [file ijms-25-10309-s001.zip › SYNPO2L novel transcript second splice donor.pdf]

SYNPO2L transcript novel second splice donor

CCTGGCTGGCAGTGGCTGCCTGAGCT
CTCTACGTAGAGACAGTTCTGACCCCTGAAGAAGCCCACCCCACTACCATCCCCAGCTTGGGGTAAATAA
TATATTTAGCTGGCGACCTTGAGGTATCCTAAAGATTTGGAGTGTTGCCTTGGAGAACAACACTCTAACT
TTTCCTGCATCTTGGATGGAGACTACCCCTGACTGTGAGTAGCAGGGGTGTGAAGCCCAGAGGACTGCCC
TGGCAGTTGGAAGGCCGAGATCAACTTTAACTTCTGTCTAAACT

GTTAGTAACAGGGTCTCTGCAGGGAGG
TCTCTGGGAAGAAGGGCCACCTAGAGCATGTAGGTGTGTGAATGTATTTGAGTGATGGTG
TTTTGATGTGCACACATGTGTATTTACATAAGTGTATGTGAATGGAGCTGTATGTAAGCT
GGGGTATATGTGAATGAGAGAGAAAAGTAAATAGGAGAAAGTACGCCTACAGTTGGCTTT
CACTAGATGGATATTCTTGTTGAAGTGCTGTGTGGTGGGAGTTATTTCTGTGAATGTGTA
TGATGTGGATTGCATGAGGAGGGTGGAATTGTGAATGAAGATCCTCATTGCTAAGGGTTC
TTTGTGCTCATATCATAGGGGTAAGGGGTAGTGGAGAGGATGAGTCCTAAGCTTCATGAG
AAGCCTCCTAACATGGTCCCTATGATGGGTGGAAAGTGTTCCTTCTCATCTTTCCTCTCC
CTGGGACCTGCTTATCTCCCTGTCTGCCACGGAGAGTGACACATCCTTCTCCTCCCCCTC
CCATTGCCAGTCAGGAATGGGAGGCAGGTGAGGAATGTGTGTGGGGTGGATAAGAGCACA
TCTCTTGGAAGATAGGAGCAGAATAGCAGTAAAATGGGATGGAAGAGCTGTGGTCAGAGC
AGTGTCAGGGCAGCCAACTGGACTCTGCCTTATCTTTAACTGTCCTTGGTTTCACCTTTC
ACCCTCCACTCTGCCCCTAAGTCAGAGGACTTTGTCACAGGGGACATTGTGGGTCGGGTC
AGTGCAAGAGGGAGCTTGCTGGGATGGGGGGTGAAAGCCTTGGGGAACAGGACTAAAAGA
GGCTGGGAAGCCCCCCTGACCATTTTACCTCTTGGCCTCCCCAG

AACTGCAACGTGCAGAGAGCCTCCAAGAGAAGAGCA
TAAAAGAGGCCAAGACCAAATGCAGGACAATTGCATCCCTGCTCACTGCAGCCCCCAACCCCCACTCCAA
AGGGGTACTTATGTTTAAGAAACGGCGGCAGAGAGCCAAGAAGTACACCCTGGTGAGCTTCGGGGCTGCT
GCTGGGACAGGCGCTGAGGAGGAGGACGGCGTTCCCCCCACGAGTGAGTCCGAGCTGGACGAAGAAGCCT
TCTCTGACGCCCGCAGCCTCACCAATCAATCTGACTGGGACAGTCCCTATCTGGACATGGAGCTTGCCAG
GGCGGGCTCAAGAGCATCAGAGGGCCAGGGCTCTGGGCTGGGAGGGCAGCTGAGTGAGGTCTCTGGGCGA
GGGGTGCAGCTCTTTGAACAGCAGCGCCAGCGCGCAGACTCCAGCACCCAGGAACTGGCACGGGTCGAAC
CAGCAGCCATGCTCAACGGGGAAGGCCTGCAGTCACCACCTCGGGCCCAGAGTGCTCCCCCAGAGGCAGC
TGTGCTCCCACCCAGCCCCTTGCCGGCGCCTGTAGCCAGCCCCAGACCCTTCCAACCAGGTGGTGGAGCC
CCGACCCCAGCTCCAAGCATCTTTAACCGGTCAGCCAGGCCCTTTACCCCGGGCCTACAAGGGCAGCGGC
CAACTACCACCTCGGTTATTTTCCGGCCTTTAGCCCCCAAAAGGGCGAACGACAGCCTGGGGGGCCTCAG
CCCCGCCCCACCCCCCTTCTTGTCTTCGCAGGGGCCCACCCCTCTGCCCAGCTTCACTTCAGGGGTTCCC
AGCCACGCGCCAGTCTCTGGTTCCCCCAGCACCCCACGCTCCTCGGGCCCTGTGACAGCCACCAGCTCCC
TGTACATCCCAGCCCCTAGTCGGCCTGTCACCCCAGGTGGAGCTCCAGAGCCCCCCGCTCCTCCTAGCGC
AGCTGCCATGACCTCCACCGCTTCTATCTTCCTATCTGCGCCTTTGCGACCCTCTGCGCGCCCAGAGGCG
CCTGCCCCAGGCCCAGGGGCTCCTGAGCCCCCCAGCGCTCGCGAGCAGCGCATCTCTGTGCCAGCTGCCC
GCACGGGTATCCTGCAGGAGGCCCGGCGCCGGGGGACCCGGAAGCAGATGTTCCGGCCGGGAAAGGAGGA
GACGAAGAACTCGCCCAACCCCGAGCTGCTATCGCTGGTACAGAACCTGGATGAAAAGCCTCGGGCCGGG
GGTGCAGAATCTGGTCCTGAAGAAGATGCTCTGAGCCTCGGGGCTGAAGCCTGCAACTTCATGCAGCCAG
TAGGGGCCAGGAGTTACAAGACCCTGCCTCACGTGACACCTAAGACCCCCCCTCCAATGGCTCCCAAGAC
CCCGCCCCCTATGACTCCTAAGACTCCACCCCCAGTGGCTCCTAAGCCCCCATCTCGAGGGCTCCTTGAT
GGGCTCGTGAATGGGGCAGCCTCTTCGGCTGGAATCCCTGAGCCACCAAGGCTGCAGGGCAGGGGTGGGG
AGCTGTTTGCTAAGCGGCAGAGCCGTGCGGACAGGTATGTGGTGGAAGGTACACCTGGTCCTGGTCTTGG
CCCTCGGCCTAGAAGTCCTTCTCCTACCCCGTCTCTGCCCCCTTCCTGGAAATATTCACCCAACATCCGT
GCCCCGCCTCCTATTGCTTACAACCCACTGCTCTCTCCCTTTTTCCCCCAGGCGGCCCGAACTCTCCCTA
AGGCCCAATCCCAGGGGCCTCGGGCAACACCCAAGCAGGGCATCAAGGCTCTAGATTTTATGCGGCATCA
GCCCTATCAACTTAAAACTGCCATGTTCTGTTTTGATGAGGTTCCCCCGACTCCTGGCCCTATCGCCTCA
GGGTCCCCCAAAACTGCCCGAGTCCAGGAGATTCGCCGGTTTTCCACTCCGGCACCCCAGCCCACTGCAG
AACCCCTGGCTCCCACTGTGCTTGCCCCCCGAGCAGCCACTACACTGGATGAGCCCATCTGGAGAACAGA
ACTGGCCTCAGCCCCTGTTCCTAGCCCAGCCCCTCCTCCAGAGGCTCCCAGGGGCCTTGGGGCTTCTCCC
AGCTCCTGCGGTTTCCAGGTAGCCAGGCCCCGATTTTCAGCCACCAGAACAGGATTGCAAGCTCATGTGT
GGAGGCCTGGGGCAGGGCACCAGTGAACAGGCACAGGTCCCAGGACCAAGGAGAGGTGGAACATCCAGTT
CCTAAAGTTGCTTCTCCTACCCTATCCCATCCCCTGTCACGCATCTGGAAGCTAAATTGCCTCCTGCCAG

Commented [JS1]: Start of novel exon

Commented [SJ2]: End of novel exon second splice donor

Commented [SJ3]: Intron for novel transcript second splice donor

Commented [SJ4]: Start of common last exon

AGATGGTTTCCAAGTTGATGTCCCCTTCCCCCACCTTCCTCCTCACTCTCTACCTCCCTGCCGCTTTCCA
ACCAAGTATGTCTGCTTTGGTATCTTTGCCTCTCTTTGTCTCTGCATTTCCTTTCCTGGATCTCTGTCTT
TATTTCCAGGCTTCTCCACCCATATTCCTCCACAGATCTCTCTTCCTTGACATTTGTGCTTTTCTCCCTG
GGCCTCATTTTAATGTTCAGTGAGAAGTAAACAGAGCAGAAGTGACCACTGGGACTTCAGGCAAGAAGCT
CACCACCAGGCACACAGCAAAGGGACTGAACTGACCCCTGTTTGCACTAAGCCACCCCCACCCCCCACTC
TGCTTTCCCAAGCTTGACTGGCATATACCTAGGCCTGTGTGTGTGTGTGTGTGTGTGTGTGTGTGTGTGT
GTGTGCTCTTCCGTCTAAGGCATGAATAAGAGGGGAGGTCAAAATAAAGACCCAATCTGAGGCCGGGCAC
GGTGGCTCACGCCGGTAATCCCAGCACTTTGGGAGGCCGAGGCGGGCGGATCACGAGGTCAGGAGATCGA
GACCATCCTGGCTAACACGGTGAAACCCCATTTCCACTAAAAATACAAAAAATTAGCTGGGCGTGGTGGC
GAGCGCCTGTAGTCCCAGCTACTCGGGAGGCTGAGGCAGGAGAATGGCATGAACCTGGAAGGCGGAGCTT
GCAGTGAGCTGAGATTGCGCCACTGCACTCCAGCCTGGGCGACGGAGCGAGACTCTGTCTCAAAACAAAC
AAACAAACAAAAGACCCAATCTGAGTCTTATCGTTGTACTGATAGAAGGGTCAGATATCCCCACATGGAG
TTGAGTGGGAGAAAGAGATTCACTAGAGAATAACTCCTTAGAGACCAATGTCTGTAGCAGGTGTACAGCA
TCTTGTGAAAGTTATGGAGCATGAAAAGACTGAAGGGCCAGGACAGTTTGCATGGGCTGAGTTATACCAG
CTAGACCAGGAATAGAACAAAGAATTCTATACCTCAGGATTTCAAAAAGTTAGCAACTTGAGAGGCCAGT
GCTGAGCAACCCAGTACCCAGGAAATGAAAAAAAGAAAGAAAATTCCCTCCGAGAATGAACAAATCATTG
GCTTCATTGCCTCATGAGCTTGAGAGAAAGGAGAAGAGAGCCAGAGTGTGGCAAGTGAGGCCAAAATCAG
AAGCATGGCAGAAATGAGTGTAAGTGATTGAGCCACAGACAGAAGTGTGGCGAGGGACAATGCCATATTG
GGAGAAGGTAAAGTTGAGTAACAAGAAACCAACCGTGTGTGAGAGGGGGATTGGAAAAAAATTTGAGGGA
GAAGAATGTTAGAATGGAAGGGAATGATGGTGGAAGGGAGGTGTGAGGGTGTGTGCTGAGTGTTGAAAGA
ACGGTTGGTGTCTGTGTGATTTTCCTTGAGTCTGTTCTTCAGTGTGTCTTCTGCAGCTTGCCATGACTGC
CTGGGAAAGAGTAGGGAAATACCCAGAGCCAAAACCTCCTTTCAGTCCCACCCCATCCCTCAAAACCCCA
GCTATTGCTTCTTTTCAGCTTCAGGTCCTGATCTCCAATCTTAGTATGGACTCCCTTCTCACCAAGACCA
CCACCAGCTACGTTTGCTGTGTAATCTGGAAAGTGATAATTTCCTTTGCTTGTTGGGTGTGAGTCACAAT
ACTTTGGTTTGTGCACAAGAATAAATTTATGCCCCATACCTTC

SYNPO2L.N2 cDNA

First exon highlighted in yellow, second exon highlighted in blue, ATG start codon highlighted in green.

CCTGGCTGGCAGTGGCTGCCTGAGCTCTCTACGTAGAGACAGTTCTGACCCCTGAAGAAGCCCACCCCAC
TACCATCCCCAGCTTGGGGTAAATAATATATTTAGCTGGCGACCTTGAGGTATCCTAAAGATTTGGAGTG
TTGCCTTGGAGAACAACACTCTAACTTTTCCTGCATCTTGGATGGAGACTACCCCTGACTGTGAGTAGCA
GGGGTGTGAAGCCCAGAGGACTGCCCTGGCAGTTGGAAGGCCGAGATCAACTTTAACTTCTGTCTAAACT
AACTGCAACGTGCAGAGAGCCTCCAAGAGAAGAGCATAAAAGAGGCCAAGACCAAATGCAGGACAATTGC
ATCCCTGCTCACTGCAGCCCCCAACCCCCACTCCAAAGGGGTACTTATGTTTAAGAAACGGCGGCAGAGA
GCCAAGAAGTACACCCTGGTGAGCTTCGGGGCTGCTGCTGGGACAGGCGCTGAGGAGGAGGACGGCGTTC
CCCCCACGAGTGAGTCCGAGCTGGACGAAGAAGCCTTCTCTGACGCCCGCAGCCTCACCAATCAATCTGA
CTGGGACAGTCCCTATCTGGACATGGAGCTTGCCAGGGCGGGCTCAAGAGCATCAGAGGGCCAGGGCTCT
GGGCTGGGAGGGCAGCTGAGTGAGGTCTCTGGGCGAGGGGTGCAGCTCTTTGAACAGCAGCGCCAGCGCG
CAGACTCCAGCACCCAGGAACTGGCACGGGTCGAACCAGCAGCCATGCTCAACGGGGAAGGCCTGCAGTC
ACCACCTCGGGCCCAGAGTGCTCCCCCAGAGGCAGCTGTGCTCCCACCCAGCCCCTTGCCGGCGCCTGTA
GCCAGCCCCAGACCCTTCCAACCAGGTGGTGGAGCCCCGACCCCAGCTCCAAGCATCTTTAACCGGTCAG
CCAGGCCCTTTACCCCGGGCCTACAAGGGCAGCGGCCAACTACCACCTCGGTTATTTTCCGGCCTTTAGC
CCCCAAAAGGGCGAACGACAGCCTGGGGGGCCTCAGCCCCGCCCCACCCCCCTTCTTGTCTTCGCAGGGG
CCCACCCCTCTGCCCAGCTTCACTTCAGGGGTTCCCAGCCACGCGCCAGTCTCTGGTTCCCCCAGCACCC
CACGCTCCTCGGGCCCTGTGACAGCCACCAGCTCCCTGTACATCCCAGCCCCTAGTCGGCCTGTCACCCC
AGGTGGAGCTCCAGAGCCCCCCGCTCCTCCTAGCGCAGCTGCCATGACCTCCACCGCTTCTATCTTCCTA
TCTGCGCCTTTGCGACCCTCTGCGCGCCCAGAGGCGCCTGCCCCAGGCCCAGGGGCTCCTGAGCCCCCA
GCGCTCGCGAGCAGCGCATCTCTGTGCCAGCTGCCCGCACGGGTATCCTGCAGGAGGCCCGGCGCCGGGG
GACCCGGAAGCAGATGTTCCGGCCGGGAAAGGAGGAGACGAAGAACTCGCCCAACCCCGAGCTGCTATCG

CTGGTACAGAACCTGGATGAAAAGCCTCGGGCCGGGGGTGCAGAATCTGGTCCTGAAGAAGATGCTCTGA
GCCTCGGGGCTGAAGCCTGCAACTTCATGCAGCCAGTAGGGGCCAGGAGTTACAAGACCCTGCCTCACGT
GACACCTAAGACCCCCCCTCCAATGGCTCCCAAGACCCCGCCCCCTATGACTCCTAAGACTCCACCCCCA
GTGGCTCCTAAGCCCCCATCTCGAGGGCTCCTTGATGGGCTCGTGAATGGGGCAGCCTCTTCGGCTGGAA
TCCCTGAGCCACCAAGGCTGCAGGGCAGGGGTGGGGAGCTGTTTGCTAAGCGGCAGAGCCGTGCGGACAG
GTATGTGGTGGAAGGTACACCTGGTCCTGGTCTTGGCCCTCGGCCTAGAAGTCCTTCTCCTACCCCGTCT
CTGCCCCCTTCCTGGAAATATTCACCCAACATCCGTGCCCCGCCTCCTATTGCTTACAACCCACTGCTCT
CTCCCTTTTTCCCCCAGGCGGCCCGAACTCTCCCTAAGGCCCAATCCCAGGGGCCTCGGGCAACACCCAA
GCAGGGCATCAAGGCTCTAGATTTTATGCGGCATCAGCCCTATCAACTTAAAACTGCCATGTTCTGTTTT
GATGAGGTTCCCCCGACTCCTGGCCCTATCGCCTCAGGGTCCCCCAAAACTGCCCGAGTCCAGGAGATTC
GCCGGTTTTCCACTCCGGCACCCCAGCCCACTGCAGAACCCCTGGCTCCCACTGTGCTTGCCCCCCGAGC
AGCCACTACACTGGATGAGCCCATCTGGAGAACAGAACTGGCCTCAGCCCCTGTTCCTAGCCCAGCCCCT
CCTCCAGAGGCTCCCAGGGGCCTTGGGGCTTCTCCCAGCTCCTGCGGTTTCCAGGTAGCCAGGCCCCGAT
TTTCAGCCACCAGAACAGGATTGCAAGCTCATGTGTGGAGGCCTGGGGCAGGGCACCAGTGAACAGGCAC
AGGTCCCAGGACCAAGGAGAGGTGGAACATCCAGTTCCAAAGTTGCTTCTCCTACCCTATCCCATCCCCT
GTCACGCATCTGGAAGCTAAATTGCCTCCTGCCAGAGATGGTTTCCAAGTTGATGTCCCCTTCCCCCACC
TTCCTCCTCACTCTCTACCTCCCTGCCGCTTTCCAACCAAGTATGTCTGCTTTGGTATCTTTGCCTCTCT
TTGTCTCTGCATTTCCTTTCCTGGATCTCTGTCTTTATTTCCAGGCTTCTCCACCCATATTCCTCCACAG
ATCTCTCTTCCTTGACATTTGTGCTTTTCTCCCTGGGCCTCATTTTAATGTTCAGTGAGAAGTAAACAGA
GCAGAAGTGACCACTGGGACTTCAGGCAAGAAGCTCACCACCAGGCACACAGCAAAGGGACTGAACTGAC
CCCTGTTTGCACTAAGCCACCCCCACCCCCCACTCTGCTTTCCCAAGCTTGACTGGCATATACCTAGGCC
TGTGTGTGTGTGTGTGTGTGTGTGTGTGTGTGTGTGTGCTCTTCCGTCTAAGGCATGAATAAGAGGGG
AGGTCAAAATAAAGACCCAATCTGAGGCCGGGCACGGTGGCTCACGCCGGTAATCCCAGCACTTTGGGAG
GCCGAGGCGGGCGGATCACGAGGTCAGGAGATCGAGACCATCCTGGCTAACACGGTGAAACCCCATTTCC
ACTAAAAATACAAAAAATTAGCTGGGCGTGGTGGCGAGCGCCTGTAGTCCCAGCTACTCGGGAGGCTGAG
GCAGGAGAATGGCATGAACCTGGAAGGCGGAGCTTGCAGTGAGCTGAGATTGCGCCACTGCACTCCAGCC
TGGGCGACGGAGCGAGACTCTGTCTCAAAACAAACAAACAAACAAAAGACCCAATCTGAGTCTTATCGTT
GTACTGATAGAAGGGTCAGATATCCCCACATGGAGTTGAGTGGGAGAAAGAGATTCACTAGAGAATAACT
CCTTAGAGACCAATGTCTGTAGCAGGTGTACAGCATCTTGTGAAAGTTATGGAGCATGAAAAGACTGAAG
GGCCAGGACAGTTTGCATGGGCTGAGTTATACCAGCTAGACCAGGAATAGAACAAAGAATTCTATACCTC
AGGATTTCAAAAAGTTAGCAACTTGAGAGGCCAGTGCTGAGCAACCCAGTACCCAGGAAATGAAAAAAAG
AAAGAAAATTCCCTCCGAGAATGAACAAATCATTGGCTTCATTGCCTCATGAGCTTGAGAGAAAGGAGAA
GAGAGCCAGAGTGTGGCAAGTGAGGCCAAAATCAGAAGCATGGCAGAAATGAGTGTAAGTGATTGAGCCA
CAGACAGAAGTGTGGCGAGGGACAATGCCATATTGGGAGAAGGTAAAGTTGAGTAACAAGAAACCAACCG
TGTGTGAGAGGGGGATTGGAAAAAAATTTGAGGGAGAAGAATGTTAGAATGGAAGGGAATGATGGTGGAA
GGGAGGTGTGAGGGTGTGTGCTGAGTGTTGAAAGAACGGTTGGTGTCTGTGTGATTTTCCTTGAGTCTGT
TCTTCAGTGTGTCTTCTGCAGCTTGCCATGACTGCCTGGGAAAGAGTAGGGAAATACCCAGAGCCAAAAC
CTCCTTTCAGTCCCACCCCATCCCTCAAAACCCCAGCTATTGCTTCTTTTCAGCTTCAGGTCCTGATCTC
CAATCTTAGTATGGACTCCCTTCTCACCAAGACCACCACCAGCTACGTTTGCTGTGTAATCTGGAAAGTG
ATAATTTCCTTTGCTTGTTGGGTGTGAGTCACAATACTTTGGTTTGTGCACAAGAATAAATTTATGCCCC
ATACCTTC

Inferred AA sequence from first ATG

METTPDCE
